# An environmental physical activity and nutrition intervention in early childhood education and care settings: process evaluation of the NAPSACC UK multi-centre cluster RCT

**DOI:** 10.1186/s12966-026-01882-4

**Published:** 2026-02-13

**Authors:** Jemima Cooper, Kim Hannam, Stephanie Chambers, Tom Reid, Russell Jago, Zoi Toumpakari, Sharon Anne Simpson, Miranda Pallan, Madeleine Cochrane, Ruth Kipping, Rebecca Langford

**Affiliations:** 1 Bristol Medical School, University of Bristol, Bristol, UK; 2 School of Social and Political Sciences, University of Glasgow, Glasgow, UK; 3 National Institute for Health and Care Research Applied Research Collaboration West (NIHR ARC West), Bristol, UK; 4 School for Policy Studies, University of Bristol, Bristol, UK; 5 MRC/CSO Social & Public Health Sciences Unit, School of Health and Wellbeing, University of Glasgow, Glasgow, UK; 6 Department of Applied Health Sciences, University of Birmingham, Birmingham, UK

**Keywords:** Obesity, Physical activity, Nutrition, Childcare, Pre-school children, Process evaluation, Qualitative, UK, Prevention

## Abstract

**Background:**

Most children attend early childhood education care settings (ECEC settings), commonly known as nurseries in the United Kingdom. ECEC settings provide opportunities to improve health through improved nutritional quality and physical activity for young children. There is evidence from the US that the NAPSACC intervention improves nutrition and physical activity in ECEC settings. We adapted NAPSACC for the UK and investigated its fidelity, acceptability and sustainability within a multi-centre trial.

**Methods:**

Embedded process evaluation within a 12-month cluster randomised controlled trial with 52 ECEC settings (25 intervention and 27 control). The NAPSACC UK intervention comprised two six-month cycles of nutrition and activity self-assessment, staff workshops and goal setting, supported by public health practitioners. Data included: observations during training and workshop delivery, questionnaires to practitioners and ECEC setting staff; 11 interviews with practitioners who delivered the intervention, 11 ECEC setting managers, 5 commissioners, and two focus groups with the research team. Document analysis of self-assessment and goal setting forms was undertaken. Thematic analysis was conducted with both deductive and inductive codes, a coding framework and triangulation across data sources.

**Results:**

Three-quarters (19/25) of intervention ECEC settings implemented the NAPSACC intervention across one cycle. Only 40% implemented a second cycle, mainly due to delays in scheduling staff workshops caused by sector-wide staffing challenges. ECEC setting managers valued the opportunity to reflect on practice and the support offered by the practitioner. ECEC setting staff highly rated the workshops and valued support given by public health practitioners. 83% of nutrition and 70% of physical activity goals set by the ECEC settings were achieved (fully or partially) and self-assessment scores increased, with greater gains for ECEC settings implementing two cycles. ECEC setting managers planned to maintain the changes made but varied in their intention to continue self-assessment and goal-setting processes.

**Conclusions:**

Despite sector-wide staffing challenges, we saw high engagement from ECEC settings in self-assessment and setting goals to improve child nutrition and activity. However, future development and use of NAPSACC UK need to be considered in the context of a lack of measurable impact on objective measures of child health and the significant challenges of staff capacity and time.

**Trial registration:**

ISRCTN33134697, 31/10/2019.

**Supplementary Information:**

The online version contains supplementary material available at 10.1186/s12966-026-01882-4.

## Background

On a global scale, young children are not meeting physical activity or dietary national recommendations [1, 2]. In the UK, only 9% of 2–4 year-olds met the recommended minimum of three hours physical activity per day [3] and 15% of 1.5–3.5 year olds consumed the recommended amount or less of free sugars [4]. Lower than recommended levels of physical activity and dietary factors, including consumption of energy-dense foods, large portions sizes and snacking contribute to a complex dynamic of biological, environmental and behavioural associations with obesity [5]. Consequently, there is a critical need to improve young children’s physical activity and diet.

Early childhood education and care settings (ECEC settings) (usually known as nurseries, pre-schools or day-care in the UK) are an important environment in which young children spend a substantial amount of time. Many governments around the world encourage attendance at ECEC settings with funding or subsidies [6–9]. In England and Scotland for example, government-funded childcare recently increased to 30h per week [10]. As such, ECEC settings are key settings to improve nutrition and physical activity in young children, yet the evidence base for ECEC setting-based health interventions remains limited.

A systematic review of strategies to support the implementation of healthy eating, physical activity and obesity prevention policies, practices or programmes in ECEC settings concluded that while these strategies may strengthen implementation, they showed little to no effect on children’s diet, activity levels or weight [11]. The evidence base for such policies and programmes remains limited as the review identified few high-quality studies to guide practice [11]. Similarly, a systematic review of 58 healthy eating programmes in ECEC settings reported small improvements in children’s diet quality but no meaningful changes in BMI, with heterogeneity between studies making it challenging to identify reasons for the lack of impact [12]. To address the limitations of the existing evidence base and heterogeneity of reported studies, rigorous evaluations, including process evaluations of implementation, are needed to build evidence on effective ECEC setting interventions for improving child health. Furthermore, process evaluations can help to unpick why interventions did not work and help us understand the importance of contextual factors for intervention implementation and impact [13, 14].

The Nutrition and Physical Activity Self-Assessment for Childcare (NAPSACC) intervention was developed in the United States to improve nutrition and physical activity in ECEC settings through modifications to the environment, policies and practices via a process of self-assessment, goal-setting and targeted technical assistance [15]. Randomised Controlled Trials (RCTs) of NAPSACC in the US have shown effectiveness across several outcomes. These include: healthier food and mealtime environments (as measured by an environmental audit nutrition score); greater staff knowledge around childhood obesity and healthy eating; small but significant reductions in children’s Body Mass Index (zBMI); and increases in accelerometer-measured physical activity [15–17]. The intervention is currently used in over 30 states across the US [18, 19].

**Fig. 1:**
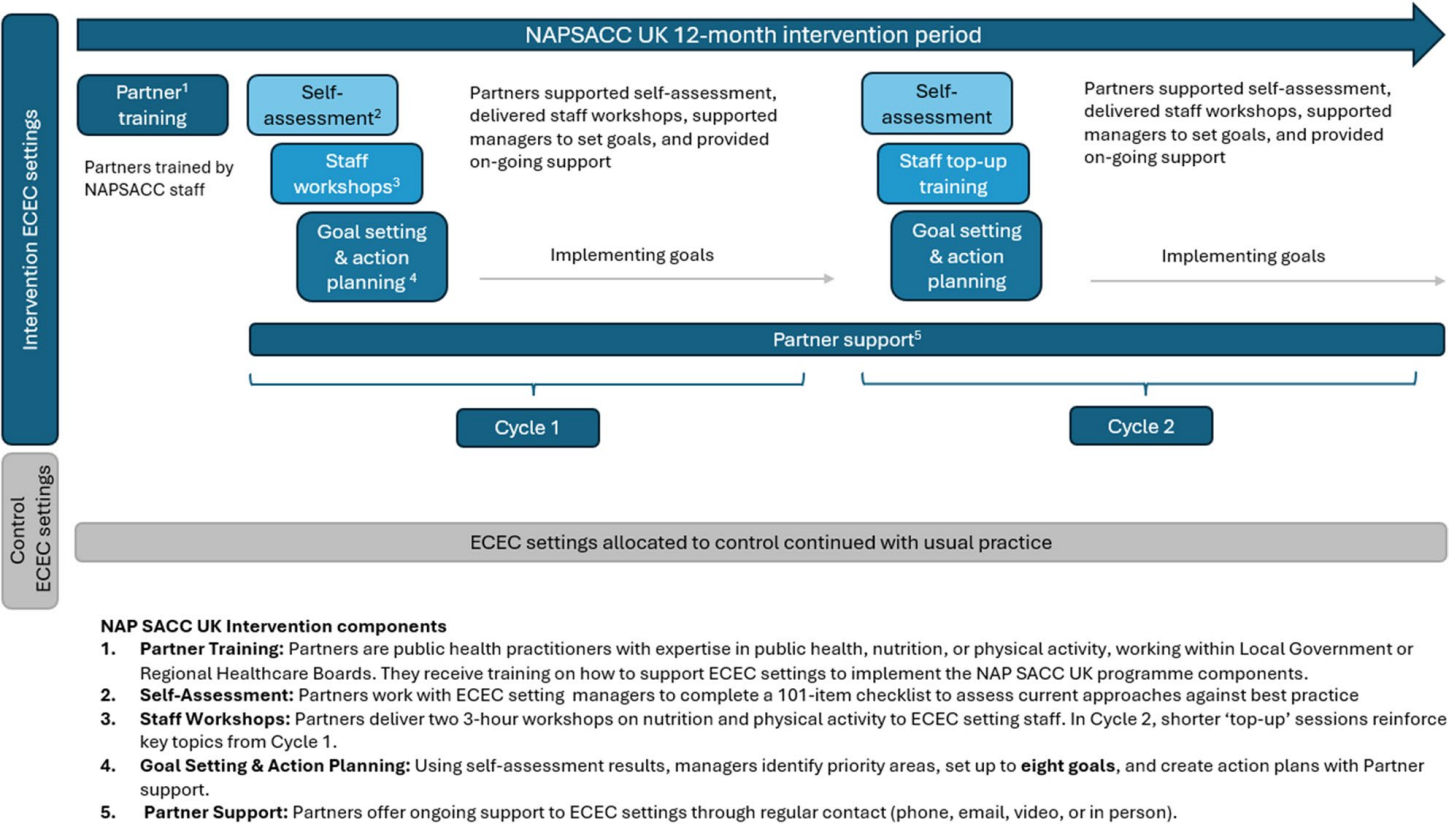
NAPSACC UK intervention

The NAPSACC intervention was adapted for the UK and a feasibility cluster-RCT was conducted in 2015-2016 [20–22]. The intervention was acceptable and feasible. A full-scale cluster-RCT [23] to evaluate the effectiveness and cost-effectiveness of NAPSACC UK commenced in 2019, seeking to improve nutritional quality and physical activity, while reducing sedentary time and portion size in line with national recommendations. Based on the US intervention, NAPSACC UK asked ECEC settings to engage in a process of self-assessment, goal setting and action planning, with support provided by trained public health practitioners (NAPSACC UK Partners). Figure 1 explains the key elements of NAPSACC UK, with further details in the study protocol [23] and TIDieR checklist (Additional file 1). Unlike the US version, NAPSACC UK incorporated a second cycle of self-assessment and goal-setting to embed reflective practices within ECEC settings, increase the intervention dose, and enhance the potential to improve child health outcomes, recognising that a single cycle may be insufficient to address all areas for potential improvement.

The trial (reported in full separately [24]) found no evidence of an intervention effect for the co-primary outcomes of average kcal per eating occasion consumed at ECEC settings (adjusted geometric mean ratio, 0·86 [0·72 to 1·03], *p* = 0·09 or minutes of total physical activity (adjusted mean difference, −2.13 [−10.96,6.70], *p* = 0.64). Lower lunch energy served ((aMD − 69·1 kcal per occasion (95%CI −116 to −22·2; *p* = 0·004)) and consumed (aMD − 67·7 kcal per occasion (95%CI −118·6 to −18·7, *p* = 0·009)) were observed in intervention settings as secondary outcomes.

To aid interpretation of the main study findings and to inform recommendations for future implementation, policy, and practice, we conducted an embedded process evaluation, informed by complex intervention and process evaluation frameworks [13, 14]. The process evaluation addressed four key research questions:

1. Was the NAPSACC UK intervention delivered with fidelity – that is, were all components (partner training, self-assessment, staff workshops, goal-setting and action-planning, goal implementation, and partner support) implemented as intended?
2. Was the NAPSACC UK intervention acceptable to ECEC setting staff and the NHS/Healthcare Board staff (NAPSACC UK Partners) who supported its implementation?
3. Were changes made because of NAPSACC UK likely to be sustainable over time?
4. How did broader contextual factors influence NAPSACC UK’s implementation and impact?

## Methods

### Study design, sample and recruitment

NAPSACC UK was a 12-month multi-centre, parallel-group, two-arm, cluster-RCT with a repeated cross-sectional design [23]. Recruitment started in 2019, paused in 2020 due to the COVID-19 pandemic, and restarted in 2022. The trial included ECEC settings from four areas across England (Somerset, Sandwell, Swindon) and Scotland (Ayrshire & Arran) selected to represent differing levels of deprivation and ethnicity.

In the UK, ECEC settings eligible to participate in NAPSACC UK included: private nurseries, maintained (Local Government-funded) nurseries, nurseries attached to primary schools, private day nurseries, or preschools, where lunch was consumed. In each study area, all eligible ECEC settings (*n* = 437) were invited to participate with consenting ECEC settings randomly allocated to the NAPSACC UK intervention or usual practice (control) group [24]. We asked intervention ECEC settings to complete two 6-month cycles of training, self-assessment and goal setting, supported by public health practitioners (‘Partners’) as outlined in Fig. 1.

### Measures

We focused on four key processes evaluation measures:

- Fidelity: were all the intervention elements implemented as intended? Also known as adherence or delivery [25], and including measures of ‘dose’ (e.g. how much of the intervention was delivered/received).
- Acceptability: were the intervention components (and resulting changes to policy/practice) acceptable to ECEC setting staff?
- Sustainability: was the reflective cycle of self-assessment and goal setting likely to be embedded into routine practice? Were the changes made as a result of the goals likely to be maintained over time?
- Context: what external factors might have acted as barriers or facilitators to implementation and/or impact?

### Data collection

We used both qualitative and quantitative methods to gain a detailed understanding of how NAPSACC UK was implemented and received, as described below and in Table 1.

**Table 1:** Summary of process evaluation methods

Method	Participants	Details	Fidelity	Acceptability	Sustainability	Context
Observations						
Partner training	Partners	All training session (*n* = 3, 2 in England, 1 in Scotland)	✔	✔		✔
Staff workshops	Intervention ECEC setting staff	12 workshops, sampled to ensure variation in site location, topic, ECEC setting size and deprivation level	✔	✔		✔
Questionnaires						
Partner training evaluation forms	Partners	15/15 evaluation forms received		✔		
Staff workshop evaluation forms	Intervention ECEC setting staff	200 evaluation forms received; denominator not recorded		✔		
Usual practice questionnaire	Control ECEC setting managers	15/27 forms received (56% response rate)				✔
Semi-structured interviews						
Semi-structured interviews	Intervention ECEC setting managers	11 (of 25) managers interviews, sampled to ensure variation in location, and ECEC setting size and deprivation level	✔	✔	✔	✔
Semi-structured interviews	Partners	11 (of 15) Partners interviewed. 3 Partners had left role and one did not respond to interview	✔	✔	✔	✔
Semi-structured interviews	Local commissioners	Local Authority or NHS Healthcare Board Staff (*n* = 5)		✔	✔	✔
Document analysis						
Self-assessment forms	−	Collected from all intervention ECEC settings at start and end of each completed cycle	✔			
Goal setting forms	−	Collected from all intervention ECEC settings at start and end of each completed cycle	✔			
Partner support logs	−	Collected from all Partners at end of intervention	✔			
Focus group						
Focus groups	Research Site Managers, Trial Manager and Project Administrator	Conducted at end of intervention with research staff to gain insights into implementation (*n* = 2 focus groups, 6 participants in total)	✔	✔		✔
Fieldnotes						
Fieldnotes	−	Fieldnotes taken throughout the study relevant to process evaluation aims	✔	✔	✔	✔

#### Observations

Fieldworkers observed all Partner training sessions (*n* = 3) and twelve ECEC setting staff workshops (sampled across all four study sites and both physical activity and nutrition topics) to explore fidelity and quality of implementation. Observations were semi-structured and included both quantitative scoring and open qualitative observations on three key areas: coverage of workshop materials, facilitator quality (knowledge, presentation and facilitation skills), and participants’ engagement. Researchers were trained prior to observations to ensure a consistent approach in both scoring and focus of qualitative observations (see Additional File 2). Coverage of specific workshop topics was scored as a binary (covered/not covered), supplemented with additional qualitative notes to explain why topics were excluded (e.g. ran out of time) or other relevant factors (e.g. additional topics covered in response to staff question). Facilitator quality and participants’ engagement levels were scored from 1 (“very poor”) to 5 (“very good”), with a detailed scoring rubric provided to ensure consistency.

#### Questionnaires

Questionnaires were developed asking Partners and ECEC setting staff to rate training elements using a score of 0–5 (see Additional File 3). Partners completed questionnaires to capture views on quality and usefulness of training in preparing them for their role. ECEC setting staff completed a similar evaluation questionnaire after each workshop. Participants completed questionnaires either on paper or online depending on training delivery. Control ECEC setting managers completed a short questionnaire at the end of the study to assess any changes to usual practice and/or potential contamination.

#### Interviews and focus groups

The lead author (JC) interviewed NAPSACC UK Partners, intervention ECEC setting managers and local commissioners in each area upon completion of their involvement in the intervention to explore their experiences and views of the NAPSACC UK programme. All Partners were invited for interview. A sub-sample of ECEC setting managers were interviewed to provide variation in terms of deprivation level, ECEC setting type, ECEC setting size, and level of engagement in NAPSACC UK (informed through conversations with local Research Site Managers (RSMs)). Local commissioners responsible for child health were identified through their original involvement in commissioning NAPSACC UK and invited to participate. Interviews took place before the results of the trial were known. JC and RL also conducted two focus groups with research staff to gather further insight into intervention implementation. ECEC setting managers were remunerated for taking part in the interviews with a £20 voucher.

Semi-structured topic guides were used to structure the conversations, specific to participant type (Partner, ECEC setting manager, commissioner or research team, Additional File 4). We conducted interviews/focus groups online via Microsoft Teams or telephone, recorded with consent and transcribed verbatim.

#### Document analysis

ECEC setting managers completed self-assessment and goal setting forms with Partner support at the start and end of each cycle. The self-assessment forms comprised 101 multiple choice questions across different categories including: child nutrition (food/drink provided, feeding environment, menus and variety, education and professional development, and policy), lunchboxes (food/drink provided, education and professional development, policy, feeding environment), physical activity and play (time provided, indoor play environment, staff practices, education and professional development, and policy), outdoor play and learning (outdoor play, outdoor physical environment, education and professional development, and policy), and screen time. See Kipping et al. [26] for more detail. Goal setting forms recorded areas for improvement and specific goals in those areas. Managers and Partners reviewed goals at the end of each cycle, noting whether they had been fully, partially or not achieved. Partner support logs recorded support given to ECEC settings throughout the intervention including mode of delivery, frequency and duration of support.

#### Fieldnotes

Process evaluation leads (RL, JC) used fieldnotes to record relevant discussions and reflection, and to record data on relevant contextual changes (see Table 2) throughout the study.

**Table 2:** Major contextual changes occurring during trial

• COVID-19 global pandemic: The coronavirus global pandemic started in 2020 and increased financial pressures on providers and instability in the childcare provider sector. The pandemic also exacerbated existing staffing issues with recruitment and retention of staff, particularly highly-qualified staff [27]. • ‘Brexit’: The UK’s decision to leave the European Union came into effect in 2020 and led to increased difficulty in recruiting new staff across the social care sector [28]. • Cost of living: The UK ‘cost of living crisis’ started in 2021 and refers to a fall in average household disposable incomes, meaning that the cost of everyday household essentials such as energy and grocery bills are increasing at a faster rate than income [29]. Since 2021 there has been an increase in the percentage of families experiencing difficulties meeting childcare costs [30]. • Increase in Government-funded free childcare hours in England and Scotland: In England, the entitlement of 30 free hours childcare for three-to-four year olds for eligible parents (those that earn less than £100,000 a year each) is being expanded to include children from nine months old in phases from April 2024 onwards with full entitlement in place from September 2025 [27]. In Scotland, the amount of funded childcare for three-to-four year olds and eligible two-year olds was expanded from 600 h to 1,140 h in August 2021 (delayed from August 2020 due to the pandemic) [6]. • Increased closure of childcare providers: Since 2015, there has been a steady decrease in the number of childcare providers in England [27]. From 2022 to 2023 there was an estimated 5% decrease in the total number of providers, and a 10% decline in the number of childminders [31], coming at a time when there has been increased demand from parents due to expansions in Government-funded free childcare hours.						

### Data management

All qualitative data were anonymised and uploaded to NVIVO 14 to facilitate data management and analysis. Partner/staff questions and documents (self-assessments, goal and support logs) were uploaded to the REDCAP data management system. We assigned anonymised IDs to interview participants; the two-letters included in quote attributions indicate research area (SW-Swindon; SS-Somerset; SD-Sandwell; AA-Ayrshire & Arran).

### Analysis

Qualitative data were analysed using thematic analysis. An initial coding framework was developed by including deductive codes derived from research questions and topic guides. JC and RL used this framework to independently code two transcripts, leading to additional inductive codes being developed. The coding framework was revised through discussion and applied to three further transcripts by JC and checked by RL. The final framework was applied to all qualitative data from transcripts, observations, and fieldnotes, with additional revisions made where necessary.

We calculated summary (mean and median) scores and standard deviations for Partner and staff evaluation questionnaire data, examining this for differences between topic (physical activity or nutrition), research area, or delivery type (in-person or pre-recorded).

Goals set by ECEC settings in each cycle were collated and mapped to the self-assessment form categories. Mean scores for each self-assessment category were calculated, as well as percentage change across mean category scores at different points (end of cycle 1, end of cycle 2).

Partner support log data were analysed using descriptive statistics.

### Ethical approval

The University of Bristol Faculty of Health Sciences Research Ethics Committee gave ethical approval for this study (Ref: 93764). Written or verbal informed consent was obtained from participants for all data collection as specified in the protocols agreed by the ethics committee.

## Results

Participants in the process evaluation included: 11 intervention ECEC setting managers (interviews); 200 intervention ECEC setting staff (post-workshop evaluation forms); 11 Partners (interviews and partner log); five Local Government or Regional Healthcare Board commissioners (interviews); 15 control ECEC setting managers (questionnaire); and document analysis of self-assessment and goal-setting from all intervention ECEC settings (see Table 1). We first present the overall fidelity of the intervention, followed by more detailed discussions of each intervention component, including acceptability, sustainability and how broader contextual factors (as summarised in Table 2) affected implementation and impact.

### Fidelity

Fifty-two ECEC settings were randomised, with 25 allocated to NAPSACC UK (Table 3) and 27 allocated to control. Six ECEC settings did not implement the intervention: four settings cited staffing issues; one setting closed following a government inspection; and one setting did not provide a reason for no implementation. Ten intervention ECEC settings (40%) completed two cycles of the intervention and nineteen (76%) completed at least one cycle. One of the twenty ECEC settings that completed the first workshops, self-assessment and goal-setting components closed meaning they did not complete Cycle 1. No ECEC settings implemented two cycles lasting the full six months, as originally intended.

**Table 3:** NAPSACC UK implementation fidelity

Partner elements		
Partner training	✔All Partners trained	
Partner support	✔All Partners provided ongoing support to ECEC settings; 318 contacts (email, phone, online or in-person meeting) over the intervention period	
ECEC setting elements		
	Cycle 1 (% implementing element)	Cycle 2 (% implementing element)
Staff workshops	Physical activity: 80% (*n* = 20/25 ECEC settings)	Top-up training: 36% (*n* = 9/25 ECEC settings)
	Nutrition: 80% (*n* = 20/25 ECEC settings)	
Self-assessment	80% (*n* = 20/25 ECEC settings)	40% (*n* = 10/25 ECEC settings)
Goal-setting	80% (*n* = 20/25 ECEC settings)	40% (*n* = 10/25 ECEC settings)

Scheduling staff workshops was challenging. Several ECEC settings were at risk of dropping out with time constraints and staffing issues preventing in-person workshops. In response, we provided pre-recorded videos covering the same content (minus interactive group elements). Consequently, nine ECEC settings completed in-person workshops, nine accessed were pre-recorded training and two received a mix of both.

To examine potential contamination, we sent questionnaires to control ECEC settings, with 15 responding (56%). Seven reported introducing physical activity initiatives (e.g. visits from sports coaches, indoor yoga, or outdoor obstacle courses) and six implemented nutrition work (e.g. weekly cooking sessions or reviewing menus) but no evidence of ‘contamination’ (e.g. sharing NAPSACC materials) was found.

### NAPSACC UK components

#### Partner training

Partners were trained August-September 2022 in hybrid in-person/online training sessions, facilitated by nutrition and physical activity (PA) experts. Two thirds (*n* = 10) attended in-person. Training covered the trial overview, staff workshops delivery, self-assessment and goal setting processes, and their role in providing on-going support.

**Fig. 2:**
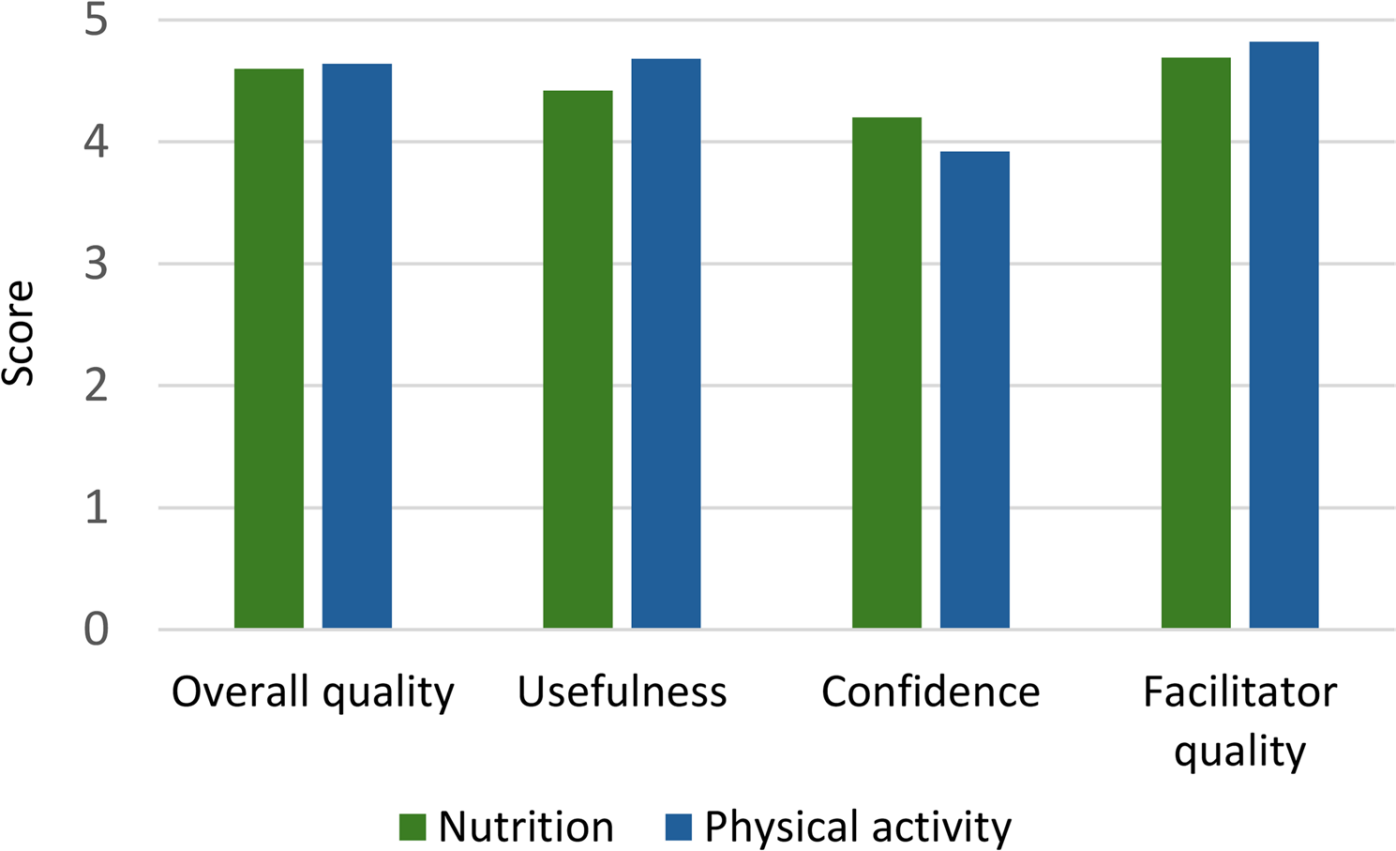
Partner assessment of training mean scores (*n* =15)

Overall, Partners rated the training highly, reporting it prepared them well for their role (Fig. 2). Observations noted Partners appeared engaged and responsive. Partners also reflected positively on training in interviews, with one noting: *“I couldn’t have delivered the program without the training”* (Partner_SD302). Partners reported lower levels of confidence after physical activity training, with some finding the content and language too technical:

*“Some of the physical health messages*,* there were technical words that I’d never heard before…. there was no way I was going to take them on and then teach somebody else.” (Partner_SS101).*

Partners found face-to-face training preferable *“because you tried the activities*,* and you could have that back-and-forth conversation”* (Partner_SD304). However, for those unable to attend in-person, online training was seen as a good alternative that did not affect learning.

#### ECEC setting staff workshops

**Fig. 3:**
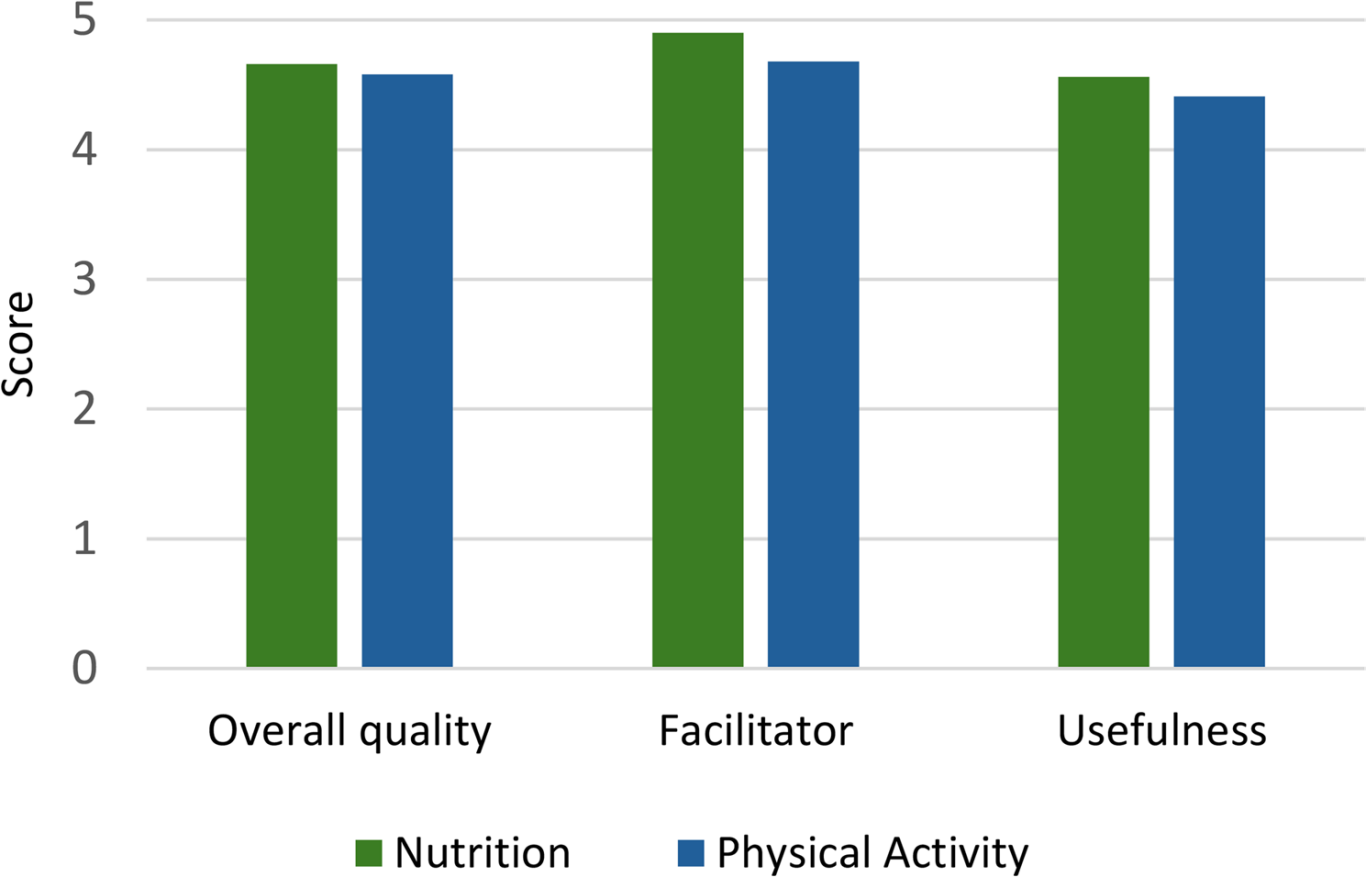
Staff evaluation of workshops mean scores (*n* = 200)

Partners delivered workshops to ECEC setting staff throughout 2022. Observation, interview, and questionnaire data indicated ECEC setting staff rated the training highly (Fig. 3). ECEC setting managers felt Partners were the right people to deliver the training, demonstrating good knowledge and expertise: *“they really knew their stuff”* (Manager_AA5023). Staff workshop questionnaires and observations indicated staff particularly valued training on portion sizes and serving practices.

We identified some minor modifications to workshop content, including shortening workshops to fit with ECEC setting timescales, and removing ‘technical’ physical activity content which Partners judged staff might struggle with: *“The language was very technical*,* I couldn’t even say some of the words… proprio[ceptor]…?*” (Partner_SS104). However, some staff (in preschools or school-based ECEC settings) reported wanting more in-depth content:

*“We already knew most of it and the bits we didn’t know*,* they tended to skim over… there was some stuff that was quite interesting*,* but they didn’t really go into depth on that… So the higher-level stuff would have been of more interest.” (Manager_SW2034)*

Consequently, some managers felt workshop content should have been tailored to each setting: *“If we met beforehand*,* we could have discussed [it]… It could have been more bespoke for us.*” (Manager_SS1041).

Staff workshops were challenging to organise; scheduling delays meant several ECEC settings only completed one cycle. Managers often struggled to free up staff for the workshops: *“I think our biggest barrier is just having that time off the floor to do things”* (Manager_SS1041). Other barriers included low staff numbers, and reluctance to ask staff to work extra hours, even if paid. These scheduling delays presented difficulties for Partners: *“There was a big gap between training and going into the nursery*,* so we then felt that we had kind of forgotten what we had learnt”* (Partner_SW201).

Staff workshops were held in-person (as intended) in nine ECEC settings with 139 staff attending. Staff reported in-person training helped encourage exchange of ideas and provided a rare opportunity for staff to meet together:

*“We don’t often get a chance to sit down together because we’re always with the children… So it did help ignite a little bit of*,* ‘we can do that*,* we can do this.’” (Manager_SS1006)*

Partners also highlighted the benefits of in-person training, particularly the opportunity to develop relationships: *“It helped build those relationships where the nursery knows what we are*,* what we do*,* who to go to if they need any support.”* (Partner_SD302).

Staff receiving pre-recorded training (*n* = 9 ECEC settings) rated it marginally lower than in-person training, but still with high levels of acceptability (Additional File 5). Managers appreciated this flexibility in training provision, noting they would have been unable to continue with NAPSACC UK without this option.

#### Self-assessment

**Fig. 4:**
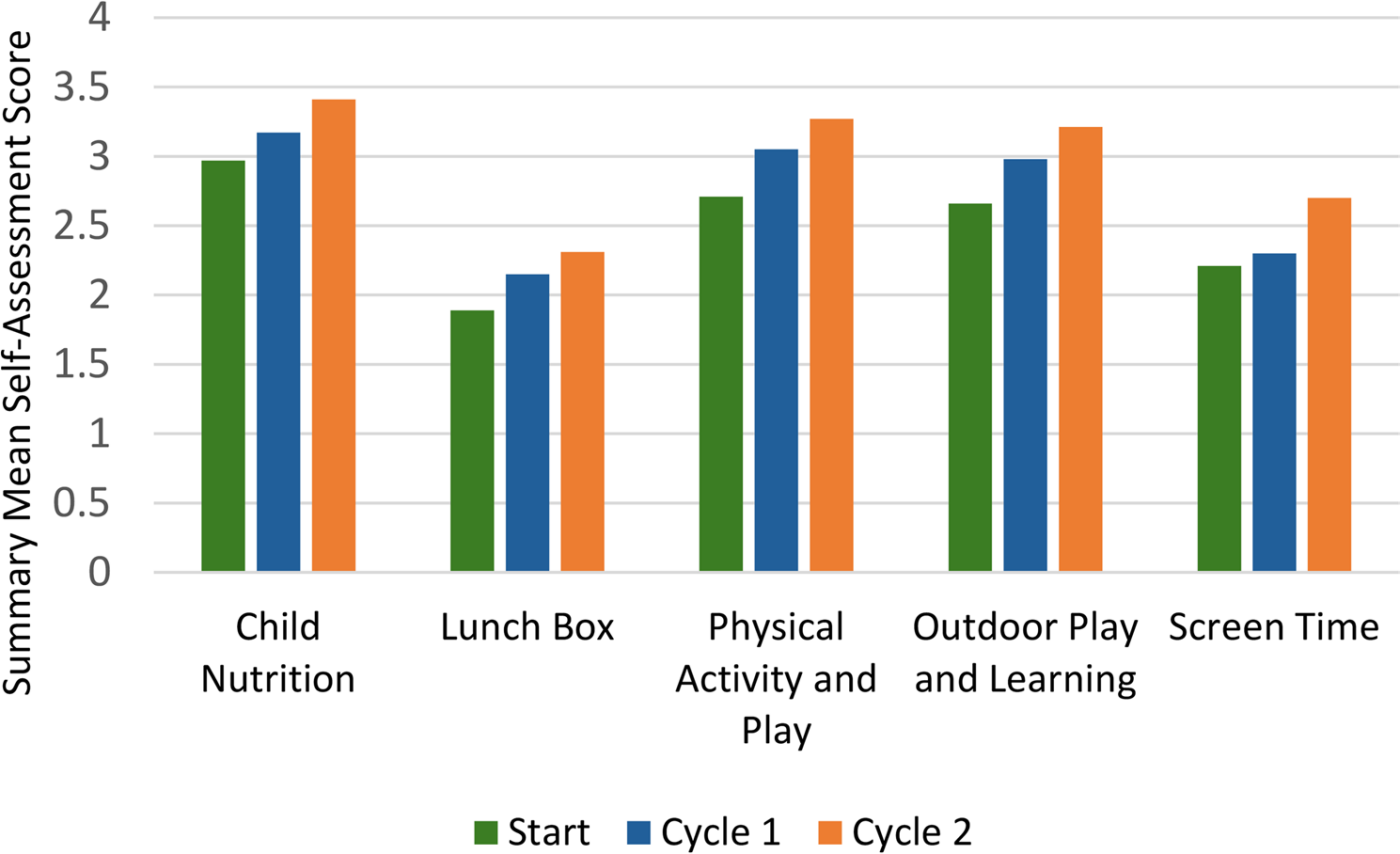
ECEC setting self-assessment summary mean scores at the start, end of Cycle 1 and end of Cycle 2

Self-assessment forms were completed by 20 ECEC settings in cycle 1 and 10 ECEC settings in cycle 2 (Table 3). Scores increased for all five assessment areas (Fig. 4), with greater increases in ECEC settings completing two cycles (Full scores presented in Additional File 6). Across all items, average scores increased by 22%. ECEC settings in the most deprived area started with lower scores but ended with similar or higher scores to the less deprived ECEC settings, indicating greater progress.

ECEC setting managers found the self-assessment form useful in identifying their strengths and areas for improvement, with one commenting *“It really made me step back and look at what we need to do as a setting”* (Manager_SD3016). Completing the assessment at the start and end of the cycle(s) helped them recognise their progress:

*“If we didn't do [the self-assessment] at the beginning, halfway through and at the end, you may not have noticed the changes because you implement them and they become normal everyday things. But when you look back, it's because of [NAPSACC] that these things have happened.” (Manager_SS1041)*

Partners similarly felt the self-assessment provided managers with a rare opportunity to reflect on current practices in a busy ECEC setting. Managers and Partners reported no issues with completing the form but felt it needed better tailoring to different ECEC settings (e.g. not all ECEC settings provided lunchtime meals).

#### Goal setting

ECEC settings were asked to set eight goals at the start of each cycle. Only 55% (*n* = 11) set eight goals in cycle 1 (range 5–8), and only 20% (*n* = 5) set eight goals in cycle 2 (range 1–8). Staff self-reported whether goals were achieved, partially achieved or not achieved. On average, 77% of goals were fully or partially achieved (83% for nutrition, 70% for physical activity) (Additional File 7).

**Fig. 5:**
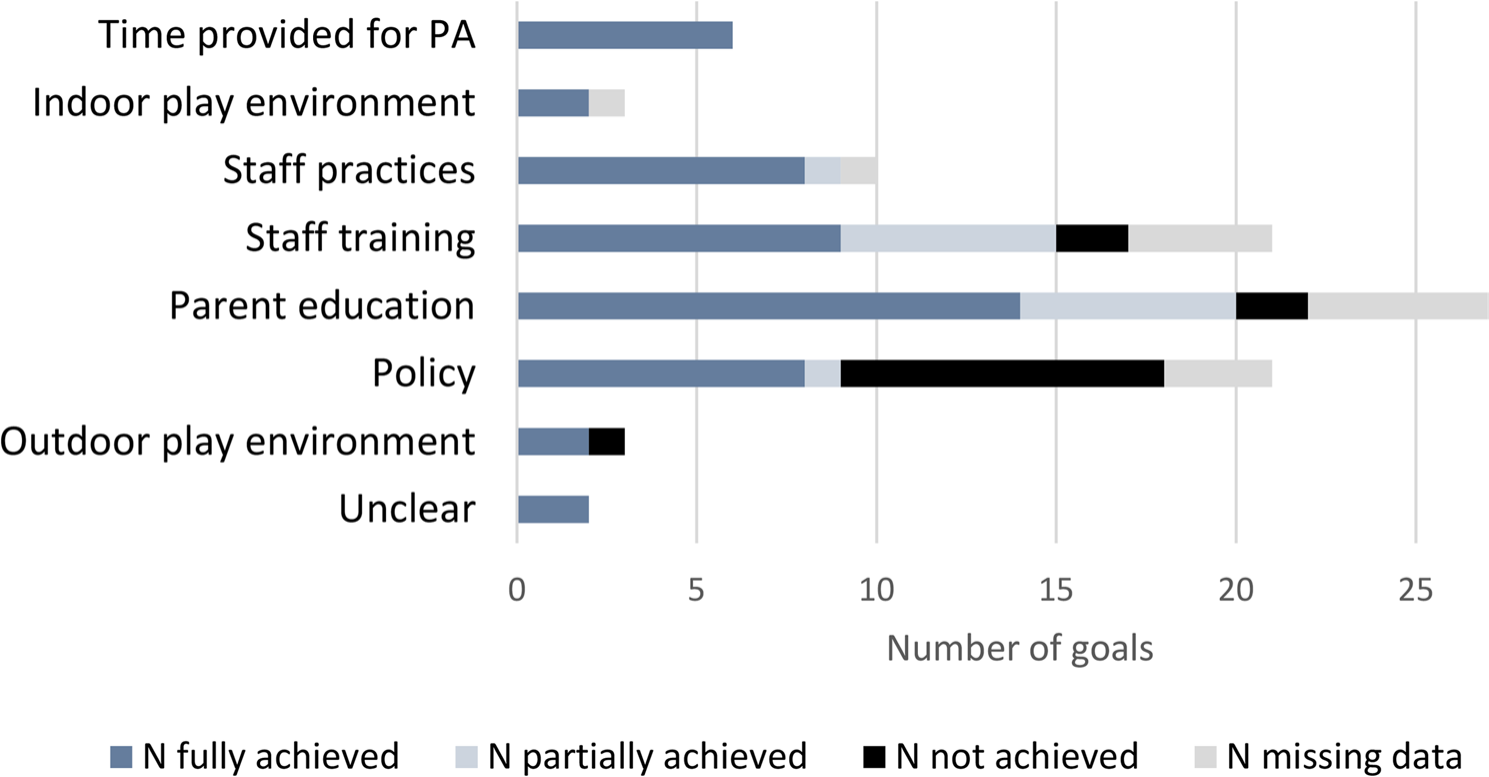
Achievement of physical activity goals across the two cycles

**Fig. 6:**
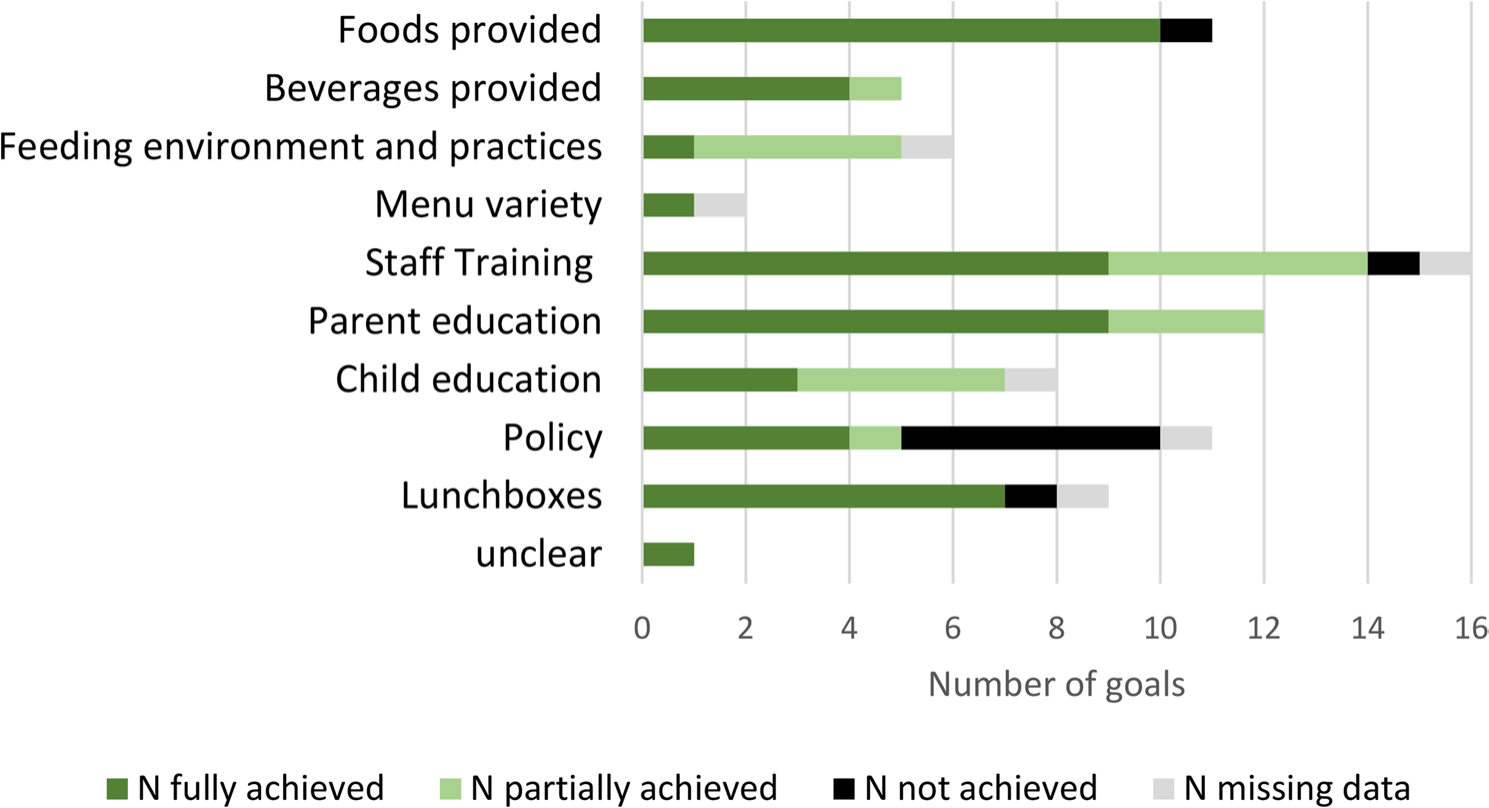
Achievement of nutrition goals across the two cycles

ECEC settings generally set goals in areas with lower self-assessment scores. For both nutrition and physical activity, most focused on increasing knowledge e.g. staff training or parent/child education (Figs. 5 and 6). Examples of these types of goals set include: ‘Staff receive professional development on child nutrition’, ‘staff to talk with child informally about healthy eating’ and ‘create a plan for professional development opportunities for staff relating to physical activity’. ECEC settings also set goals to write/update around policies (e.g. physical activity, nutrition or screentime). Examples of such goals include: ‘written policy on child nutrition’ and ‘written policy to be developed for outdoor play/physical activity’. Notably policy goals were least likely to be achieved, with managers struggling to find time or encountering barriers to approval:

*“When you implement the policy it’s gotta go to head office. It’s gotta go to head [teacher] and then it’s gotta go to governors. And then when they have a meeting that then needs to be approved*,* then it can go through.” (Manager_SS1041)*

While most goals were at least partially achieved, managers felt staff capacity sometimes limited progress. Staff shortages in settings were common and meant managers had to prioritise maintaining safe staff-child ratios rather than *“getting together to brainstorm ideas”* about how to achieve and implement goals (Manager_SD3016). A lack of time and financial resources to implement changes (exacerbated by the cost of living crisis and expansion of government-funded hours) were other common barriers across all types of goals. Where settings did have time and experienced staff members available, this facilitated goal achievement.

The goal-setting experience varied across cycles. Some ECEC settings struggled to set additional goals in cycle 2, feeling they were doing well in most areas and had already made key changes in cycle 1: *“The second goal setting is harder because they’ve done a lot of it… [they] were scrabbling around to find goals*” (Partner_SS104). However, other ECEC settings saw it as an opportunity to build on progress: *“When we set the second lot*,* we could take [it] up to the next level*,* make it bigger and fully implement it*” (Manager_SS1041).

ECEC setting staff mostly set goals as a team, with both managers and Partners feeling the staff workshops and self-assessment helped with that process. During their training, Partners were encouraged to help managers set “realistic” goals within a “sensible timeframe” (unpublished training materials). However, many goals lacked specificity, indicating a need for further training to set goals for both Partners and managers.

#### Partner support

On average, Partners contacted each ECEC setting 12 times, mostly via email. We observed no differences in level of Partner support by ECEC setting deprivation level. ECEC setting managers found the Partner support useful but varied in how much they drew on this. One manager valued knowing the support was there but had not used it much (Manager SW2034). In contrast, others found Partner support crucial in making the intervention feasible:

*“The Partner was a massive part of me carrying on with the study. When she said you’ve got to do targets and I was just like*,* ‘I don’t have time!’. My staff were working to the max!’…But once she simplified it and we looked through it*,* it was not as challenging as I first thought.” (Manager_SS1050)*

Partners supported ECEC settings by assisting with the self-assessment and goal setting, signposting to local resources or national guidance, and generally “*keeping them on track”* (Manager_AA4020).

However, Partners needed on-going support (beyond their initial training) to fulfil their role. Partners valued support from the NAPSACC UK research team in resolving any issues or ECEC setting queries. Partners also found it helpful to connect with other local NAPSACC Partners: *“Just being able to have that network of people on the same project helps a lot because you can just discuss things*” (Partner_SD303).

A significant challenge for Partners was managing their workload. All Partners had to incorporate NAPSACC into their existing role, meaning they struggled to prioritise NAPSACC activities. Their own team staff shortages created additional pressures: *“We’ve lost three members of staff that haven’t been replaced. It’s been difficult… for me to fit NAPSACC in as well as the increased workload” (*Partner_SS104). All Partners agreed any future implementation of NAPSACC would require protected time to properly engage in the role.

### Sustainability

Most managers reported they would sustain the changes made due to NAPSACC UK. They noted positive changes to improve children’s nutrition and activity, such as improving mealtime practices, encouraging activity in all weather, and staff role modelling healthy behaviours to children. Policy goals were less often achieved, but where implemented, managers expected them to continue.

Managers had mixed views on continuing with the self-assessment and goal setting. Some managers found them useful tools for planning and reflection, while others felt unlikely to use them again unless shortened and tailored to their setting.

Managers felt that staffing issues (including turnover, shortages, and illness) were a significant barrier to implementing and sustaining NAPSACC UK. Staffing issues were experienced by all ECEC settings, but particularly affected smaller ECEC settings: *“we’re a small team as well so when we’re one person down it makes a big impact”* (Manager_SS1006). Though staffing challenges were not new, managers felt they had worsened across the sector, exacerbated by the Covid-19 pandemic:

*“We were short staffed when we started it and we’re still short staffed now. It’s becoming more and more challenging but that’s how early years is at the moment.” (Manager_SS1041)*

Several Partners felt NAPSACC UK should be better targeted to ECEC settings in higher-need areas, with some Partners questioning if the ‘right’ ECEC settings had been reached. Some enrolled ECEC settings were, in their opinion, already excelling in many areas and may not have needed the intervention:

*“The nurseries we were going into were already doing very good things and perhaps weren’t in those pockets of deprivation that we should be in” (Partner_SW201).*

These Partners suggested using local knowledge to better target ECEC settings who would most benefit NAPSACC UK support, rather than those participating as *“a tick box exercise to put another credit on their front page”* (Partner_SS101).

### Commissioners’ views

Overall, commissioners felt the NAPSACC UK aligned well with their strategic focus on enhancing health and wellbeing in early years through upskilling existing staff due to budgetary constraints. Commissioners saw NAPSACC as filling a gap in early years support and saw the autonomy given to ECEC settings as a key strength. Commissioners also emphasised the need for effective, evidence-based, and cost-effective interventions:

*“If it shows an evidence base and effectiveness then that’s what we’re all quite desperate for really*,* in terms of early years settings” (Commissioner_11).*

Commissioners acknowledged a need for a more coordinated effort between Local Authorities and ECEC setting providers to improve early years health. Commissioners valued the relationships NAPSACC UK fostered between Local Government/Healthcare Board staff and early years practitioners, and felt this co-operation would be sustained. Echoing Partner views, commissioners recognised a need to allocate limited resources strategically by targeting ECEC settings where most in need. Commissioners in two areas (Somerset and Sandwell) felt they lacked the infrastructure to embed programmes like NAPSACC UK, suggesting further investment in early years support would be needed to expand NAPSACC UK to more ECEC settings.

## Discussion

NAPSACC UK was implemented with high fidelity during the first cycle, but with moderate-to-low fidelity for the intended two-cycle dose. In the first cycle, 76% of ECEC settings completed the self-assessment, implemented staff training, and set and implemented goals. However, only 40% of centres completed all these components in the second cycle. Staff availability and scheduling issues for staff workshops led to delays, preventing some ECEC settings from completing two cycles. Despite this, engagement was high: all Partners completed their training, and 80% of ECEC settings engaged with self-assessment, goal setting and workshops during cycle 1. Most goals were achieved (77%), and self-assessment scores improved, with greater gains for those completing two cycles and ECEC settings from more deprived areas. The intervention was well received by Partners and ECEC setting staff. Most managers were keen to maintain changes but suggested the self-assessment tool could be better tailored to different ECEC settings. However, Partners felt their role would not be feasible without protected time. The intervention was implemented amid a childcare crisis in the UK with staff shortages and increased ECEC setting closures [32]. We reflect below on what the process evaluation tells us about implementing NAPSACC in the UK and how our findings can help interpret the main study results. Table 4 summarises the lessons learned from implementing NAPSACC UK.

**Table 4:** Lessons learned from NAPSACC UK

• Provide ECEC settings with protected time to reflect on practice NAPSACC UK self-assessment and goal setting tools could be incorporated into annual review practices
• Prioritise in-person training where possible In-person training provides valuable opportunities to build collective practice and commitment
• But capitalise on the benefits of online training too Online pre-recorded training could be used to induct new staff into the ‘NAPSACC’ ethos
•Streamline self-assessment to increase relevance Tailor the self-assessment content to each ECEC setting by removing irrelevant content
• Encourage specific, measurable, achievable goals Use the self-assessment to support ECEC settings to set specific and measurable goals to improve physical activity and nutritional quality
• And goals that go beyond increasing knowledge Providing information alone won’t drive behaviour change. Goals should target both downstream changes (increasing education and knowledge on healthy diets and physical activity) and upstream changes (changing policy or physical environments)
• Provide protected time for Partner support Allowing Partners protected time within their job role to provide support to ECEC settings
• Focus on ECEC settings with greatest need Where time and resources are limited, focusing attention on ECEC settings with children from more deprived backgrounds may help address inequalities.

ECEC setting managers valued the protected time for reflection afforded by the self-assessment and goal setting processes. However, in busy ECEC settings, completing this process every six months was unfeasible. An annual process linked to other yearly planning activities may be more feasible. The assessment form itself also needs better tailoring to ensure relevance to ECEC settings with different provisions.

Despite being an environmental intervention, ECEC settings mostly set goals targeting staff or parent education and often lacked specificity (e.g. ‘professional development on nutrition’ or ‘give information to parents’). While most of these goals were achieved, it is well-established that information alone is unlikely to drive behaviour change [33] and undertaking training or providing information to parents may not have resulted in actual or sustained changes to practice needed to impact child outcomes. Research suggests tackling childhood obesity requires both *downstream* (education/behaviour change) and *upstream* (policy/environmental) approaches [34]. Some ECEC settings attempted *upstream* policy changes, but these were least likely to be achieved due to time constraints and bureaucratic procedures, especially in school-based settings or ECEC setting chains. Additionally, Partners may need further training on helping ECEC setting managers set ‘SMART’ (specific, measurable, achievable, realistic and time-bound) goals [35] to facilitate change.

Training was highly valued by ECEC setting staff but was challenging to schedule. In-person training developed staff enthusiasm and strengthened ECEC setting-Partner relationships. However, recorded training also proved acceptable, with broadly comparable quality ratings. Qualitative comments from managers and Partners did not suggest differing levels of enthusiasm or commitment depending on type of training received (in-person vs. pre-recorded), but we were unable to formally test this. In the US, NAPSACC has been optimized into an exclusively online, self-directed format (known as GoNAPSACC), with training moved online and partner support provided remotely via ‘technical assistance’ [36]. A randomized pilot study found this approach effective, with results suggesting the online format can drive changes in ECEC setting environments and practice [36]. High staff turnover in the UK’s early years sector is a potential threat to the sustainability of NAPSACC practices. While in-person training offers benefits in developing shared practice, inducting new staff into the ‘NAPSACC ethos’ via recorded training may offer a potential solution, but warrants further testing.

The Partner support role requires further careful consideration. ECEC settings valued the support offered, but Partners struggled to balance NAPSACC within their already-stretched existing roles. They also required additional support (beyond their initial training) to understand their role, and how best to support ECECs.

The process evaluation findings provide evidence for self-reported improvements in physical activity and nutrition provision, practices and policies in early years ECEC settings. The intervention also proved feasible for ECEC settings in areas of high deprivation, who made significant gains without need of additional Partner support. However, the main trial found no evidence these changes increased child physical activity or reduced overall energy consumption (although there was evidence of fewer calories consumed at lunchtimes) [24]. One reason for this may be the lower-than-intended intervention ‘dose’ received: only 40% of ECEC settings implemented two full cycles as intended. This lower dose is largely explained by the challenging implementation: sector-wide staffing shortages and Partner work pressures delayed initial staff workshops, leaving many without enough time to implement a second cycle. ECEC settings implementing two cycles showed greatest self-assessment improvements, suggesting benefits to repeating the process. However, the original US-based NAPSACC intervention included only one cycle, with evidence of improvements in child zBMI [16].

Another reason for the lack of impact may be that ECEC settings set goals unlikely to measurably improve physical activity or energy intake. Most goals targeted staff (or parent) knowledge, with far fewer focused on actions that might directly impact child outcomes, such as changes to the food provided or increased opportunities for activity. Additionally, policy-based goals which would influence practice across the setting were least likely to be implemented. The areas in which goals were set, while improving quality, may have been less directly related to frequency or intensity of physical activity. Similarly, many areas of nutrition targeted by NAPSACC UK were related to broader aspects of nutrition and feeding (such as staff eating meals with the children, cooking skills) which were not measured in the main trial outcomes.

It is interesting to note however, the main trial reported lower lunch energy servings and consumption in the intervention arm [24]. Free-text questionnaire comments and workshop observations suggested ECEC setting staff were particularly influenced by training on appropriate portion sizes for young children. The change observed in lunchtime energy consumption may result from staff changing portions sizes and checking satiety before offering additional servings

### Strengths and limitations

Our multi-method analysis comprehensively assessed the feasibility, acceptability, sustainability and implementation context of NAPSACC UK. We spoke to key actors (managers, Partners, researchers and commissioners) to understand how it was implemented, with analysis conducted blind to main trial outcomes. Triangulation was used to identify confirmatory or contradictory results by comparing different data sources: observations, questionnaires, document analysis and interviews. We found strong congruence in findings obtained via different methods, lending credibility to our findings. For example, ECEC setting managers’ positive views of the staff workshops were consistent with the high ratings reported in staff questionnaires. Likewise, some Partners acknowledged omitting certain workshop content, which was confirmed through workshop observations.

Three managers could not be interviewed due to capacity issues. Instead, relevant information was gathered from Partner interviews and discussions with the research staff and Trial Manager. One Partner did not respond to an interview request, but we obtained relevant information from another Partner working with the same ECEC settings. We did not include parent perspectives on NAPSACC UK. As NAPSACC is an environmental intervention targeting ECEC settings we feel this was justified but acknowledge parent interviews could have provided valuable insights into the changes observed in home-provided lunchboxes. We were able to record how many staff were given access to the pre-recorded training, but not how many accessed it. However, we received over 200 evaluation forms for the pre-recorded training suggesting substantial staff engagement. The achievement of goals (fully, partially, not achieved) was self-reported by ECEC managers and Partners and is therefore subjective. Finally, mapping goals set by ECEC settings to the self-assessment categories was both difficult and, to an extent, subjective. Other researchers may have mapped the goals differently, but this is unlikely to have changed our overall conclusions.

## Conclusions

Despite unprecedented pressures in the early years sector, engagement with NAPSACC UK was high. ECEC settings reported improvements in nutrition and physical activity provision, practices and policies. Most goals were achieved and self-assessment scores increased in all areas. However, the intervention did not improve children’s physical activity or total energy consumption [24]. This may be due to a lack of dose (nine ECEC settings completed only one cycle) or because goals often focused on increasing knowledge rather than specific behavioural changes. Given the lack of impact on child health outcomes and the challenges of staff capacity and time, broader policy-level and statutory changes, such as free lunches with mandated nutritional standards, may be more effective in improving child health and addressing inequalities.

## Supplementary Information

Supplementary Material 1.

## Data Availability

Data for the NAPSACC UK study is available upon request after approval with a signed data access agreement (https://data.bris.ac.uk/data). The study protocol was published in the BMC public health and is available at: (https://bmcpublichealth.biomedcentral.com/articles/10.1186/s12889-023-16229-y). The statistical analysis plan is available at: ([Nutrition and Physical Activity Self-Assessment for Child Care (NAPSACC UK Trial): Statistical Analysis Plan - University of Bristol] (https://research-information.bris.ac.uk/en/publications/nutrition-and-physical-activity-self-assessment-for-child-care-na) and the health economics analysis plan is available at: ([HEALTH ECONOMICS ANALYSIS PLAN (HEAP) NAPSACC UK - University of Bristol](https://research-information.bris.ac.uk/en/publications/health-economics-analysis-plan-heap-nap-sacc-uk)).

## Abbreviations

BTC: Bristol Trials Centre
ECEC setting(s): Early Childhood Education and Care setting(s)
NAPSACC: Nutrition and Physical Activity Self-Assessment for Childcare
NIHR: National Institute for Health and Care Research
RCTs: Randomised Controlled Trials
RSMs: Research Site Managers
