# Supplementary material for: An environmental physical activity and nutrition intervention in early childhood education and care settings: process evaluation of the NAPSACC UK multi-centre cluster RCT
Source: Int J Behav Nutr Phys Act. 2026 Feb 13;23:26. doi: 10.1186/s12966-026-01882-4 (PMC13014976; doi:10.1186/s12966-026-01882-4)
Supplement: Supplementary file 1 — Supplementary Material 1. [file 12966_2026_1882_MOESM1_ESM.docx]

# Additional files

- Additional file 1: TIDieR description of NAPSACC UK (.pdf)
  - A description of the NAPSACC UK intervention presented according to the TIDieR template for intervention description and replication.
- Additional file 2: Observation Guidance: Staff workshops (.pdf)
  - Observation Guidance designed to assist the Research Site Managers in undertaking the semi-structured observations of NAP SACC staff workshops, specifically regarding rating of facilitators or participant responsiveness
- Additional file 3: Partner and staff evaluation forms (.pdf)
  - Evaluation forms for partners and staff to evaluate their training and workshops
- Additional file 4: Topic guides (.pdf)
  - Topic guides for each of the interview types (Local Government/Health Board Leads/Partners/Intervention Nursery Managers)
- Additional file 5: Table showing staff training scores (in-person vs online) (.pdf)
  - This table presents data on the staff training scores for both in-person and pre-recorded training
- Additional file 6: Self-assessment scores full table (.pdf)
  - This table presents data on the self-assessment summary categories across each cycle and the percentage change in summary score across the cycles
- Additional file 7: Physical activity and nutrition goal achievement (.pdf)
  - This figure presents the percentage of goals by level of achievement for both physical activity and nutrition

### Additional file 1: TIDieR description of NAPSACC UK

| Item | Description |
| --- | --- |
| Name | Nutrition and Physical Activity Self-Assessment for Child Care UK (NAPSACC UK) |
| Why | NAPSACC UK is an intervention delivered in child care settings with the aim of improving the nutrition and physical activity environment, through a process of self-assessment and targeted assistance. NAPSACC UK is a theory-based program that employs components of social cognitive theory (SCT) and the socio-ecological framework. The objectives of the programme are to improve the nutritional quality, variety and quantity of food served, amount and quality of physical activity, staff-child interactions and staff behaviours around nutrition and physical activity and child care provider policies. |
| What: materials | The NAPSACC UK intervention is based around a self-assessment tool completed by ECEC managers with advice and support from a NAPSACC UK “Partner”. This document, called the ‘Review & Reflect’, is an 101-item multiple choice questionnaire, completed by the ECEC manager, covering areas in nutrition, physical activity and play, outdoor play and learning, and screen time.  Following completion of the Review & Reflect, the ECEC manager along with the NAPSACC UK Partner agree on eight goals; three nutrition, three physical activity and a further two of the setting’s choice. |
| What: procedures | The NAPSACC UK intervention is a five stage process:  1. Self-Assessment.  2. Workshop delivery: Specialised staff deliver workshops to all ECEC staff on: i) Nutrition; ii) Physical Activity.  3. Goal setting and Action Planning: The NAPSACC UK Partner works with the ECEC manager to develop an action plan, listing eight goals for improvement.  4. Tailored technical assistance: NAPSACC UK Partner continues regular contact with ECEC to provide support and advice toward them meeting their goals.  5. Evaluate, revise, repeat. The Review & Reflect self-assessment is repeated by the ECEC manager after six months and reviewed with the NAPSACC UK Partner to see where improvements have been made or not, and to explore ways to overcome barriers; action plans are revised to set eight new goals for the next six months. |
| Who provided | NAPSACC UK Partners and Local Authority/Health Board staff who deliver the ECEC workshops will be chosen locally from a range of health or health improvement staff with appropriate skills. All staff will be provided with one day of training led by specialists in nutrition and physical activity who provided the training in the feasibility study. The partners will deliver the intervention in addition to their Local Authority role. |
| How | The main part of the intervention will be delivered face to face; this includes Partners going through the Review & Reflect, action planning and attending or delivering the workshops (depending on whether the Partners are also the staff delivering the workshops). Other parts of the intervention, such as on-going support and advice from the NAPSACC UK Partner can be provided over the phone, by email or face to face. All parts of the intervention will be delivered to participating ECEC settings individually. Some parts may be delivered on a one-to-one basis (e.g. ECEC manager and NAPSACC UK Partner setting goals), while other parts such as the workshops will be delivered to a group of staff from one ECEC. Partners will have four days contact with each ECEC over the 12 months. |
| Where | The NAPSACC UK intervention is delivered in the ECEC itself. The NAPSACC UK Partner offers visits to the ECEC and the workshops take place at the ECEC or an online recording. |
| When and how much | The NAPSACC UK intervention takes place over 12 months. The length of the workshops are a total of six hours where they are delivered in person, followed by an online refresher workshop after 6 months; recorded workshops (without group interaction) will be available where individual staff need flexibility to engage with the workshops.The ECEC settings receive ongoing regular support over the 12 months. |
| Tailoring | The technical assistance offered by the NAPSACC UK Partner will depend on the goals. |
| Modifications | In the feasibility study the intervention was five months; in the full trial it will be 12 months. NAPSACC was designed in the US to be for a year and this longer period enables a mid-intervention review of progress against goals and further goals to be sets. In the feasibility study the Partners were Health Visitors; in the full trial Local Authorities will chose appropriate health staff. |

### Additional file 2: Observation Guidance: Staff workshops

NAP SACC UK Trial

Observation Guidance: staff workshops

This guidance is designed to assist the Research Site Managers in undertaking the semi-structured observations of NAP SACC staff workshops, specifically regarding rating of facilitators or participant responsiveness.

1. Aim of the observations

The NAP SACC UK feasibility trial identified the staff workshops as being particularly important in engaging and enthusing staff to make changes in the nursery setting. However, in the feasibility study all workshops were delivered by three physical activity and nutrition experts. In the full trial of NAP SACC we are using a different model of delivery whereby the NAP SACC UK Partners (who come from a range of professional backgrounds) will be trained up to deliver the workshops. The purpose of the observations is to gather information on how well the workshops were delivered and received to help us understand the impact of the intervention.

The observations aim to answer the following questions:

- Did the workshops cover what they were supposed to?
- Were they delivered with ‘quality’?
- How did the staff respond to the workshops?
- Were there any problems or things that worked particularly well?

The observations focus on three main areas:

- Coverage of the workshop materials
- The quality of the facilitators (in terms of knowledge, presentation and facilitation skills)
- The response from participants

The observation schedule combines quantitative (ratings) and qualitative (free text) elements to important information on how the workshops were implemented.

1. Coverage of materials

Did the facilitator cover all the intended elements of the workshop?

| 0 = No, topic not covered | 1 = Yes, topic covered |
| --- | --- |

Note: training topics may not be delivered in the same order each time. You should familiarise yourself with the intended workshop topics in advance to identify if all topics have been covered.

Training should be flexible so facilitator may bring up new issues in response to participant needs or what is suitable for their nursery location. Please note any additions to the planned training in the appropriate box (see below).

| Were any other topics covered which weren’t originally planned? Please describe (including why they were included, if known e.g. in response to participant question) |
| --- |

Any further comments should be added to the ‘additional observations/comments’ box.

| Additional observations/comments |
| --- |

Comment here if any topics missed entirely (and why that was if known e.g. training session for nursery with no outdoor space might not talk about outdoor activities).

- If some topics are covered but in a brief or superficial way, note it here, including reasons for why this was if known (e.g. ran out of time)
- Include any other relevant comments in relation to course content and coverage

1. Quality of facilitator

Below we have provided some general descriptions of what ‘very poor’ ‘adequate’ and ‘very good’ categories for each domain might look like. I have not provided specific descriptions for ‘poor’ and ‘good’ as meaningful written descriptions of each of these grades can be difficult to provide, and ultimately can be counterproductive if people use them too literally. Please use **common sense** to grade facilitators as ‘poor’ or ‘good’ if they generally fall in between the descriptions provided below. Remember we are looking for an ‘overall rating’ of the facilitators’ performance for the different sections of the workshop.

**NB the importance of adding comments:**

Please remember to add comments in the appropriate boxes to justify why you have given the facilitator a particular score. This is especially important if you give a very low or very high rating. Remember: these workshops will be delivered by people with different professional backgrounds and different knowledge bases. We need to know if this model of training people from different backgrounds works, so please add as much detail in your comments as possible.

Knowledge

| **1 = Very poor:**  Significant lack of knowledge of most of the workshop topics.  **What might this look like?**  Does not know workshop material well. Unable to answer participants questions or gave factually incorrect information. | **2 = Poor:** | **3 = Adequate:**  Adequate knowledge of majority of workshop topics.  **What might this look like?**  Appears knowledgeable on most of the workshop materials but knowledge may be somewhat limited and/or superficial. Able to answer most participants’ questions | **4 = Good:** | **5 = Very Good:**  Excellent knowledge of workshop topics.  **What might this look like?**  In-depth knowledge of workshop materials and able to draw on other knowledge in response to participant comments/questions. Able to confidently answer all participants questions. |
| --- | --- | --- | --- | --- |

Presentation skills

| **1 = Very poor:**  Poor communication and delivery of information  **What might this look like?**  Extreme nervousness, disorganised, delivery that was hard to follow, faltering or too quick to take in. Little engagement or interaction with participants. | **2 = Poor:** | **3 = Adequate:**  Adequate communication and delivery of information but lacking in some areas  **What might this look like?**  Sometimes lacking clarity of explanations, or failing to convey interest or enthusiasm for topic | **4 = Good:** | **5 = Very Good:**  Excellent communication and delivery of information.  **What might this look like?**  Presented in a clear and engaging manner. Clear explanations, delivered at suitable pace and level for all participants. Excellent engagement and interaction with participants. |
| --- | --- | --- | --- | --- |

Facilitation skills

| **1 = Very poor:**  Does not facilitate group effectively.  **What might this look like?**  Unable to adapt materials to participants’ needs; dismissive or defensive interaction with participants; unable to encourage all participants to take part; unable to control activities and keep people on task. | **2 = Poor:** | **3 = Adequate:**  Adequate facilitation of workshop:  **What might this look like?**  Some elements done well, while other areas require improvement. (Make note of what worked well/not well in comments box) | **4 = Good:** | **5 = Very Good:**  Excellent facilitation or workshop.  **What might this look like?**  Able to enthuse and engage all participants; adapts workshop materials to participants’ needs; ensures appropriate pace/level of communication; effectively manages group dynamics; provide clear guidance and structure; provides appropriate support when needed. |
| --- | --- | --- | --- | --- |

1. Participant Responsiveness

Assesses how participants respond to the training course (i.e. the extent to which participants appear interested and engaged with different aspects of the training). NB The rating is conducted for the responsiveness of the ***whole group***. (If a minority are disengaged, note this in the comments sections).

As before, don’t forget to **add comments** to justify your ratings!

Note that a rating of **‘very poor’** is for individuals who are **actively** disengaged and may express dissatisfaction with the training content or facilitators themselves. A rating of **‘poor’** should be used for more **passive** expressions of disinterest/lack of engagement (e.g. checking phone a lot during workshop, lots of ‘off topic’ chat, not wanting to take part). Because of this important distinction I have provide a description for each of the 5 points on the scale (rather than just for very poor / adequate/ very good, in previous examples).

Note that some individuals may ask a number of questions and challenge the instructor. If this is done in a constructive manner and it appears that individuals are seeking further insight then the responsiveness can be rated as ‘good’/’very good’. If individuals are challenging in a manner that appears destructive and disruptive then the responsiveness can be rated as ‘very poor’.

| **1 = Very poor:**  Participants were very uninterested and/or hostile to the facilitator or workshop materials.  **What would this look like?**  Frequently talking to other participants (not about workshop topics) while facilitator is presenting; muttering, tutting, scoffing, eye-rolling; aggressively challenging or interrupting facilitator; refusing to take part in activities. | **2 = Poor:**  Participants were uninterested in the workshop materials  **What would this look like?**  Participants gazing off into space, frequently using their phones, often talking to other participants (not about workshop topics), not responding to facilitator’s questions, little enthusiasm for taking part in activities**.** | **3 = Adequate:**  Some engagement with facilitator and workshop materials but not overly enthusiastic.  **What would this look like?**  Participants appear to be listening for the most part, but few questions asked, few comments relating the workshop content to their own nursery context and/or practice. Will take part in group activities but reluctantly, may need encouragement. | **4 = Good:**  Participants were interested in the workshop materials and engaged positively with materials and activities.  **What would this look like?**  Participants are actively interested in the materials, willing to ask questions and seek clarification. Happy to take part in activities. | **5 = Very Good:**  Participants enthusiastically engaged with the workshop materials and activities.  **What would this look like**?  Lots of verbal and non-verbal indications of interest (e.g. nodding heads, mummurs of agreement or taking notes). Enthusiastic interaction with materials (e.g. people asking questions, relating content to own nursery context and/or practice). Enthusiastic participation in group activities. Laughter, joking, having fun (while remaining on-topic) |
| --- | --- | --- | --- | --- |

1. Improvements/Problems

The final section of the form is for you to reflect on any elements that went well or not so well. (It’s OK to repeat things here which you may have mentioned in previous sections).

There is also a section to note any particular problems or issues that affected the delivery of the workshop. This could be any number of things but examples might include:

- Equipment failure
- Facilitator losing their voice
- Room too hot or cold
- Fire alarm going off in middle of session
- Someone hurting themselves during the session e.g. twisting an ankle
- Atmosphere in the nursery (e.g. something may have happened that day which is distracting the staff – perhaps notification of an Ofsted visit)

Please use the final box to add in any other comments you feel may be relevant. Remember, the more detail the better so **if in doubt, write it down!**

### Additional file 3: Partner and staff evaluation forms

# **NAP SACC UK PARTNER EVALUATION FORM**

Please let us know what you thought about today’s training workshop. Your feedback will help with the evaluation of the NAP SACC UK trial. By filling in this form we will assume you consent to us using this anonymised data in our evaluation. Thank you.

1. Which area do you work in? (Please circle)

| Ayrshire & Arran | Sandwell | Swindon | Somerset |
| --- | --- | --- | --- |

1. What is your job title (Please circle)

| Health Visitor | Dietician |
| --- | --- |
| Health Improvement Officer | Zing Team Member |
| Other (please state) ­­­­­­­­­­­­­­­ ____________________________ | |

1. How would you rate the **overall quality** of the training? Please **circle** your choice.

| Very poor Very good | | | | |
| --- | --- | --- | --- | --- |
| 1 | 2 | 3 | 4 | 5 |

1. Was the length of training…? Please **circle** your choice.

| Too short | About right | Too long |
| --- | --- | --- |

1. How useful did you find the following topics for your work on the NAP SACC UK study? Please **circle** your choice.

|  | Not useful Very useful | | | | |
| --- | --- | --- | --- | --- | --- |
| Session 1: add topic here | 1 | 2 | 3 | 4 | 5 |
| Session 2: add topic here | 1 | 2 | 3 | 4 | 5 |
| Session 3: add topic here | 1 | 2 | 3 | 4 | 5 |
| Session 4: add topic here | 1 | 2 | 3 | 4 | 5 |
| Session 5: add topic here | 1 | 2 | 3 | 4 | 5 |

Please add comments to explain your ratings (e.g. the usefulness may relate to your previous knowledge or how the training topics were covered):

|  |
| --- |

1. Please rate the quality of the trainers. Please **circle** your choice.

| **NUTRITION TRAINER** | Very poor Very good | | | | |
| --- | --- | --- | --- | --- | --- |
| knowledge of the subject | 1 | 2 | 3 | 4 | 5 |
| presentation skills | 1 | 2 | 3 | 4 | 5 |
| interaction with participants | 1 | 2 | 3 | 4 | 5 |
| **PHYSICAL ACTIVITY TRAINER** | Very poor Very good | | | | |
| knowledge of the subject | 1 | 2 | 3 | 4 | 5 |
| presentation skills | 1 | 2 | 3 | 4 | 5 |
| interaction with participants | 1 | 2 | 3 | 4 | 5 |

Please add comments to explain your ratings:

|  |
| --- |

1. How confident do you now feel about working with the nurseries on the NAP SACC UK programme? Please **circle** your choice.

| Not at all confident Very confident | | | | |
| --- | --- | --- | --- | --- |
| 1 | 2 | 3 | 4 | 5 |

1. What were the most important things you learned from this training?

|  |
| --- |

1. Is there anything you think we should change about this training?

|  |
| --- |

1. Are there any topics you think that are missing from this training?

|  |
| --- |

Many thanks for your help.

Please post this to the University in the envelope provided.

# **NAP SACC UK Workshop Evaluation Form**

# **PHYSICAL ACTIVITY WORKSHOP**

Please let us know what you thought about today’s workshop. Your feedback will help with the evaluation of the NAP SACC UK trial. By filling in this form we will assume you consent to us using this anonymised data in our evaluation. Thank you.

| Nursery Name | |  | | | | | | | | |
| --- | --- | --- | --- | --- | --- | --- | --- | --- | --- | --- |
| Location (please circle) | | Ayrshire & Arran | | Sandwell | | | Swindon | | | Somerset |
| Date of workshop | |  | | | Time of workshop | | |  | | |
| What age children do you **most often** work with in the nursery. **Circle** all that apply | | | | | | | | | | |
| Under 1 year old | 1 year olds | | 2 year olds | | | 3 year olds | | | 4 year olds | |
|  |  |  |  |  |  |  |  |  |  |  |

1. On a scale of 1-5, please rate how useful you found the following topics (**circle** your choice).

|  | Not useful Very useful | | | | |
| --- | --- | --- | --- | --- | --- |
| Workshop topics to be added | 1 | 2 | 3 | 4 | 5 |
| Workshop topics to be added | 1 | 2 | 3 | 4 | 5 |
| Workshop topics to be added | 1 | 2 | 3 | 4 | 5 |
| Workshop topics to be added | 1 | 2 | 3 | 4 | 5 |
| Workshop topics to be added | 1 | 2 | 3 | 4 | 5 |
| Workshop topics to be added | 1 | 2 | 3 | 4 | 5 |
| Workshop topics to be added | 1 | 2 | 3 | 4 | 5 |

1. One a scale of 1-5, please rate the quality of the workshop (**circle** your choice).

|  | Very poor Very good | | | | |
| --- | --- | --- | --- | --- | --- |
| Trainer’s knowledge of the subject | 1 | 2 | 3 | 4 | 5 |
| Trainer’s presentation skills | 1 | 2 | 3 | 4 | 5 |
| Trainer’s interaction with participants | 1 | 2 | 3 | 4 | 5 |
| Overall quality of the workshop | 1 | 2 | 3 | 4 | 5 |

1. Was the length of training…? Please **circle** your choice.

| Too short | About right | Too long |
| --- | --- | --- |

# **PLEASE TURN OVER**

What were the most important things you learned from this workshop?

Is there anything you will do differently in the nursery as a result of this workshop? If so, what?

Is there anything you think we should change about this workshop?

Many thanks for your help! Please post this to the University in the envelope provided.

# **NAP SACC UK Workshop Evaluation Form**

# **NUTRITION WORKSHOP**

Please let us know what you thought about today’s workshop. Your feedback will help with the evaluation of the NAP SACC UK trial. By filling in this form we will assume you consent to us using this anonymised data in our evaluation. Thank you.

| Nursery Name | |  | | | | | | | | |
| --- | --- | --- | --- | --- | --- | --- | --- | --- | --- | --- |
| Location (please circle) | | Ayrshire & Arran | | Sandwell | | | Swindon | | | Somerset |
| Date of workshop | |  | | | Time of workshop | | |  | | |
| What age children do you **most often** work with in the nursery. **Circle** all that apply | | | | | | | | | | |
| Under 1 year old | 1 year olds | | 2 year olds | | | 3 year olds | | | 4 year olds | |
|  |  |  |  |  |  |  |  |  |  |  |

1. On a scale of 1-5, please rate how useful you found the following topics (**circle** your choice).

|  | Not useful Very useful | | | | |
| --- | --- | --- | --- | --- | --- |
| Workshop topics to be added | 1 | 2 | 3 | 4 | 5 |
| Workshop topics to be added | 1 | 2 | 3 | 4 | 5 |
| Workshop topics to be added | 1 | 2 | 3 | 4 | 5 |
| Workshop topics to be added | 1 | 2 | 3 | 4 | 5 |
| Workshop topics to be added | 1 | 2 | 3 | 4 | 5 |
| Workshop topics to be added | 1 | 2 | 3 | 4 | 5 |
| Workshop topics to be added | 1 | 2 | 3 | 4 | 5 |

1. One a scale of 1-5, please rate the quality of the workshop (**circle** your choice).

|  | Very poor Very good | | | | |
| --- | --- | --- | --- | --- | --- |
| Trainer’s knowledge of the subject | 1 | 2 | 3 | 4 | 5 |
| Trainer’s presentation skills | 1 | 2 | 3 | 4 | 5 |
| Trainer’s interaction with participants | 1 | 2 | 3 | 4 | 5 |
| Overall quality of the workshop | 1 | 2 | 3 | 4 | 5 |

1. Was the length of training…? Please **circle** your choice.

| Too short | About right | Too long |
| --- | --- | --- |

# **PLEASE TURN OVER**

What were the most important things you learned from this workshop?

Is there anything you will do differently in the nursery as a result of this workshop? If so, what?

Is there anything you think we should change about this workshop?

Many thanks for your help! Please post this to the University in the envelope provided.

### Additional file 4: Topic guides

**NAP SACC UK: Local Government/Health Board Leads Topic Guide**

**Your role**

| 1. Tell me a bit about your role within the Local Authority/Health Board (LA/HB). | ·     Remit and responsibility  ·     Probe responsibility for early years and child health |
| --- | --- |

**Local context**

| 2. Tell me a bit about the local area you are responsible for. | ·     Geographical patch  ·     Demographics (e.g. deprivation levels, ethnic make-up etc) |
| --- | --- |
| 3. What are the local priorities for child health in your area? | ·     How are these priorities determined? |
| 4. What’s already happening in your area around childhood obesity prevention? | ·     Do you feel what is currently offered is enough/appropriate? |
| 5. Your LA/HB agreed to take part in NAP SACC originally back in 2018/19. It’s now 2023/4. What has changed in the past 5-6 years that might have affected NAP SACC? | · Changes in local priorities, national government, staff within the LA/HB, other early years programmes in the area, Brexit, COVID, cost of living crisis, expansion of childcare provision, shortage of staff, any other significant contextual changes.  · Recent reports indicating worsening child health, especially in under 5s |
| 6. What do you feel are the best the routes of communication to Early Years settings around updated guidance/research for Early Years health (particularly PA and nutrition)? |  |



**Involvement in NAP SACC UK**

| 7. Do you know the history of why your LA/HB decide to take part in NAP SACC? | ·     How does it fit with current local policies? |
| --- | --- |
| 8. The LA/HB provided the staff to work as NAP SACC UK Partners in supporting the nurseries. How do you think this went overall? | · Probe positive and negative experiences  - E.g. +ve - opportunities for staff development/career progression, meeting local priorities (any other opportunities coming off the back of NAPSACC e.g. additional work with nurseries as a result?)  - E.g. -ve - managing existing staff workloads, staff skills, capacity and capabilities, unintended consequences  - How it fits with other work they’re doing locally  - Delivering something so prescriptive? |
| 9. What do you think the benefits of being involved in NAP SACC have been? | ·     For LA/HB  ·     For NAP SACC UK Partners  ·     For Early Years settings |
| 10. What have been the challenges of being involved in NAP SACC? | ·     For LA/HB  ·     For NAP SACC UK Partners  ·     For Early Years settings |

**Future**

| 11. From your experience so far would you continue using NAP SACC in your local area? | · If no, why not? (prompt on budget, staffing constraints, existing interventions, etc)  · Is there the budget and would you be willing to continue to cover the cost of staff to deliver NAPSACC?  · Would you be willing to pay for a licence for the intervention (any other experience of this)?  o This could be around 23,000 pounds based on current US license fee costs  o Would include access to NAPSACC resources (website) and training and support  · Would you still want access to a university NAPSACC team to support and advise the implementation and evaluation of the programme?  · Do you think nurseries in your area will want to take part in NAPSACC? (recruitment potential)  · Who would be the most appropriate staff to deliver it in the future? |
| --- | --- |
| 12. Would you recommend it to other LA/HBs? | · Why? Any caveats?  · Are there some areas you think it would work better than others? |
| 13. What factors are most likely to affect the funding or commissioning of a programme like NAP SACC UK? | · Locally, nationally?  · Do LA/HB have resources to commission externally or will it only ever work by using their staff? |
| 14. Do you feel NAPSACC fits well with local policies on improving child health moving forwards? | · Could NAPSACC help you achieve progress towards local child health improvement goals? |

**Closing**

| 15. What would be the best way to disseminate the findings of the study? | • In your local area?  • To other local authorities or health boards elsewhere?  • What are the most important things to get across?  Best methods/opportunities to use? |
| --- | --- |
| 16. Is there anything else you would like to tell me about the study? | |
| 17. Do you have any questions for me? | |

**NAP SACC UK: Partner Topic Guide**

About you

| 1. Tell me a bit about your professional background and experience | - Briefly probe previous experience/knowledge of - Physical activity - Nutrition - Early years |
| --- | --- |
| 1. How did you get involved in NAP SACC UK? | - How/why they got involved - How it fits with their (wider) role |

Training and support

| 1. How did you find the initial Partner training at the start of the intervention (physical activity and nutrition)? | - Was it useful? - Did it cover the right things, at the right level? - How did it work being in-person/online/mixed? - How well prepared did you feel for delivering workshops and supporting nurseries? |
| --- | --- |
| 1. How useful was the online top-up training (with Kim and Ruth)? | - What did they get from it? - Was it necessary? |
| 1. Did you have to ask the NAP SACC team for additional info or support? | - What sort of questions did you have? - Were the team able to help? - Did you look for information or resources elsewhere? - What other information would have been useful? |
| 1. Did you discuss your work with other NAP SACC partners? | - What sort of things did you discuss? - Would regular meetings between Partners have been helpful? |
| 1. What additional support do you think Partners would need if NAP SACC was rolled out? | - Direct support (e.g. online forum? Specific NAPSACC contact point? More training?) - Indirect support (e.g. better accommodation of the role within current commitments?) |

Nurseries

| 1. Tell me about the nurseries you worked with. | - How many did you work with? - Nursery context (demographics, deprivation level, available resources) - Relationship they developed with manager/staff   - Has there been a nursery manager change?   - And how has that affected NAP SACC implementation? - Were some easier to work with than others? Why? - Did you feel the staff were engaged with the programme? Which bits? |
| --- | --- |

Workshops

| 1. How did you find trying to set up the workshops with your nurseries?   (Ask relevant questions below) | - What made it challenging/straight forward? - Was there a delay? Why? - Did they do in-person/online/none? |
| --- | --- |
| 1. IN PERSON TRAINING: | - When did this happen? - How many staff attended? - Did you feel comfortable delivering the training sessions? - Did it cover the right topics? Was it relevant to nursery staff? Was it at the right level? - What went well/less well? - Level of staff engagement - Did it affect your relationship with staff? - If delayed, how did this affect delivery of NAP SACC and goals? |
| 1. ON-LINE TRAINING: | - Why did they opt for online training? - When did this happen? - How was this implemented? (Did staff watch together or individually?) - Did you get any feedback from manager/staff on what they thought of the training? - Did you notice any difference between nurseries engaging in in-person or online training? |
| 1. NO TRAINING: | - Why did the nursery not do any training? - How did this impact the goal setting and engagement with NAP SACC? |

Review & Reflect

| 1. How did you find using the R&R with the nursery managers? | - When did this happen? - Did they complete it with manager? Online or in-person? - Was it a useful exercise for the nursery? - Any problems? - Differences between cycle 1 and cycle 2 (if both cycles completed)? |
| --- | --- |

Goals

| 1. Tell me about the sorts of goals the nurseries set in cycle 1 | - Why were they chosen? - How much progress was made? - Do you feel that the progress recorded on the goal setting document was accurately reflected what happened in the nursery setting? - Facilitators/barriers to progress? |
| --- | --- |
| 1. What sorts of goals did they set in cycle 2 (if applicable)? | - Why were they chosen? - Was process of choosing them different from cycle 1? - Easier or harder to achieve than cycle 1? Why? - Was it useful having two cycles? |
| 1. If they didn’t do a second cycle… | - Why didn’t this happen? - What impact did this have on their progress? - What impact might it have had if it went ahead? |
| Particular issues to explore | - Ability to influence menus (e.g. if external catering company) - Staff issues (for Partners themselves; for nursery staff) - Monitoring of lunchbox contents - Cost of living and impact on food prices - Impact of covid - Any other issues identified during the course of the trial |

Nursery support

| 1. Tell me about how you offered ongoing support to nurseries | - What sort of support did you offer? - How often and in what format (email, phone, video call, in person?) - Did you provide nurseries with extra resources (other than NAPSACC resources)? What and why? - Did some nurseries require more support than others? Why? - Did you feel able to support nurseries adequately? |
| --- | --- |

Future

| 1. How have you found working on the NAP SACC project? | - What did you enjoy the most? - What were the biggest challenges? |
| --- | --- |
| 1. How well did it fit with your current role? | - Complementary or in tension with other parts of role? - Fit with skills and experience? |
| 1. How much time did your NAP SACC work take? | - More or less than you expected? - What was most time-consuming? |
| 1. Would it be sustainable for NAP SACC to become part of your role? | - What would need to happen for this to be possible? |
| 1. Is there anything you would change about the NAP SACC programme? | - Things to include or take out? |

Closing

| 1. Is there anything else you would like to tell me about the study? |
| --- |
| 1. Do you have any questions for me? |

**NAP SACC UK: Intervention Nursery Managers Topic Guide**

Nursery context

| 1. Tell me about your role as manager in the nursery | - Are they full time? Are they also the owner? - If part of a chain, explore their relationship with management and other nurseries - If part of a school, explore their relationship with the school management. How separate or integrated are they? - Has there been a nursery manager change? - And how has that affected NAP SACC implementation? |
| --- | --- |
| 1. Why did you decide to take part in NAP SACC UK? | Any physical activity or nutrition related issues you were already concerned about with your children |
| 1. Have you been involved in any other major initiatives since starting NAPSACC | - What did this/these initiatives focus on? - Did these affect your participation in NAP SACC UK in any way? |

**Remind manager of the NAP SACC elements (R&R, workshops, goal setting, Partner support) and explain we want to get their views on each of these, as well as the overall impact NAPSACC has had in their nursery.**

Review & Reflect

| 1. How did you find completing the Review and Reflect form? | - Who did you complete it with (and when)? - Was it useful? What did it highlight? - Any problems? - Differences between cycle 1 and 2? (If applicable) |
| --- | --- |

Staff workshops

| 1. When did your staff workshops take place? | - If delayed, explore why and what impact this had. |
| --- | --- |
| 1. What did you think of the physical activity and nutrition NAP SACC workshops offered to your staff? | - Were they useful? How? - Any problems? - Staff reactions to the workshops |
| 1. [If relevant] How was the Partner in delivering the workshops? | - Quality of training sessions - Benefits/drawbacks of being in-person - Were they the most appropriate person to deliver the workshops? (Personality, level of knowledge) |
| 1. [If relevant] Tell me about the online training? | - Why did they do it online? - Did staff watch together or online? - Quality of online training - Staff reactions/engagement with online workshops - Benefits/drawbacks of being online |
| 1. [If relevant] How useful was the online top-up training? | - How well was this completed by staff (if known)? - Was the top-up session needed? |
| 1. How were staff compensated for attending the out-of-hours training? | - TOIL, overtime? - Was the manager happy to accommodate this? - Do they have a specific training budget? - How did they cover staff absences if done during worktime? |
| 1. Additional resources: Did you look for or use any other resources or forms of training to help you? | - What did they use? - Why was this needed? - What extra training or resources might have been useful? |
| 1. **If the nursery didn’t do the training** | - Why did the training not happen? - Did they consider doing the online training? - What impact did not doing the training have? - Did the staff have enough knowledge to implement the goals? |

Goal setting

| 1. Tell me about the goals you set in cycle 1.   (SRA to remind them of what goals they set) | - Why did you choose them? - Who helped set them (Partner? Staff? Owner?) - **How much progress did you make?** - **What helped or hindered your progress?** - How engaged were the staff in delivering these goals? |
| --- | --- |
| 1. Tell me about the goals you set in cycle 2. | - Why did you choose them? - How much progress did you make? - How engaged were the staff in delivering these goals? - Easier or harder to achieve than cycle 1? Why? - Was it useful having two cycles? |
| 1. If they didn’t do a second cycle… | - Why didn’t this happen? - What impact did this have on their progress? - What impact might it have had if it went ahead? |
|  |  |
| 1. We asked you to come up with both physical activity goals and some nutrition goals | - was it easier to set goals in one area than the other? - was there a difference in how easy or hard it was to make changes in these two areas? |

Partner support

| 1. Tell me about the contact you had with your NAP SACC UK Partner. | - Was it useful? - How often and what format was the contact? - Were they the right person to offer support? Could they answer all your questions? - Were there any problems? - Did the support differ between cycle 1 and cycle 2? |
| --- | --- |

Overall reflections

| 1. What sort of impact (if any) has NAPSACC had in your nursery? | - children’s physical activity and diet - staff practices - policies, structures and the nursery environment - Relationship with the Partner - Engagement with parents - Anything else |
| --- | --- |
| 1. What have been the biggest benefits of NAP SACC for your nursery | - (if not already mentioned in above question) |
| 1. What have been the biggest challenges? | - Probe staffing issues, cost of living crisis and Covid/Brexit - Constraints if part of school/chain or have external catering company - **For SCOTLAND – impact of Scottish funding** - Any other issues? - Any unintended consequences? |
| 1. Will you be able to sustain the changes you’ve made now that the study has ended? | - Facilitator or barriers - How might staff turn-over affect this? |
| 1. Will you continue to use the Review & Reflect and goal-setting process? | - When and how often? |
| 1. Where do you receive/look for updated guidance/research around Early Years health currently (particularly PA and nutrition)? How would you like to hear about this? |  |
| 1. Is there anything you would change about the programme? | - Things to add in or take away? |

Recruitment (ask if you have time)

| How did you find recruiting children to take part in the study? | Was this difficult? Why?  What could have helped this? |
| --- | --- |

Closing

| 1. Is there anything else you would like to tell me about the study? |
| --- |
| 1. Do you have any questions for me? |

### Additional file 5: Table showing staff training scores (in-person vs online)

| Training workshop | Delivery mode | Attendance (in-person)  Given access (online) | Number completing eval form | Overall quality of training | Facilitator quality | Usefulness of training |
| --- | --- | --- | --- | --- | --- | --- |
| Nutrition | Overall | N/A | 96 | 4.66  Median: 5  Range: 2 (3-5)  IQ: 1 | 4.90  Median: 5  Range: 2 (3-5)  IQ: 0 | 4.56  Median: 5  Range: 4 (1-5)  IQ: 1 |
|  | In-person | 74 | 62 | 4.85  Median: 5  Range: 1 (4-5)  IQ: 1 | 4.90  Median:  Range:  IQ: | 4.67  Median: 5  Range: 4 (1-5)  IQ: 1 |
|  | Online | 112 | 34 | 4.30  Median: 4  Range: 2 (3-5)  IQ: 1 | N/A | 4.35  Median: 5  Range: 3 (2-5)  IQ: 1 |
| Physical Activity | Overall | N/A | 86 | 4.58  Median: 5  Range: 2 (3-5)  IQ: 1 | 4.68  Median: 5  Range: 2 (3-5)  IQ: 1 | 4.41  Median: 5  Range: 3 (2-5)  IQ: 1 |
|  | In-person | 65 | 65 | 4.68  Median: 5  Range: 2 (3-5)  IQ: 1 | 4.68  Median: 5  Range: 2 (3-5)  IQ: 1 | 4.44  Median: 5  Range: 2 (3-5)  IQ: 1 |
|  | Online | 112 | 21 | 4.25  Median: 4.5  Range: 2 (3-5)  IQ: 1.25 | N/A | 4.28  Median: 4  Range: 3 (2-5)  IQ: 1 |
| Top-up | Online | 76 | 18 | 4.44  Median: 4.5  Range: 2 (3-5)  IQ: 1 | N/A | 4.30  Median: 4  Range: 2 (3-5)  IQ: 1 |

### Additional file 6: Self-assessment scores full table

| *Self-assessment summary scores* | |  |  |  |  |  |  |
| --- | --- | --- | --- | --- | --- | --- | --- |
|  | | Cycle 1  (n=20) | Cycle 2  (n=10) | Intervention end (n=19) | % change - overall  (n=19) | % change - completed 1 cycle (n=9) | % change – completed 2 cycles (n=10) |
| Child Nutrition Summary Score | | 2.97 (0.32) | 3.17 (0.30) | 3.41 (0.27) | 14.6 (10.6) | 12.1 (7.9) | 16.8 (12.6) |
|  | Food provided | 2.88 (0.46) | 2.96 (0.48) | 3.14 (0.43) | 10.0 (14.6) | 7.3 (10.9) | 12.4 (17.5) |
|  | Beverages provided | 3.76 (0.27) | 3.87 (0.17) | 3.95 (0.12) | 5.4 (8.5) | 4.7 (11.1) | 6.1 (5.9) |
|  | Feeding environment | 3.37 (0.24) | 3.54 (0.27) | 3.58 (0.24) | 6.4 (9.2) | 5.7 (3.9) | 7.0 (12.4) |
|  | Menus and variety | 3.12 (0.93) | 3.33 (0.87) | 3.35 (0.79) | 2.6 (13.5) | -3.1 (8.8) | 8.3 (15.4) |
|  | Nutrition education and professional development | 2.70 (0.65) | 3.34 (0.39) | 3.59 (0.38) | 41.4 (37.6) | 31.1 (21.8) | 50.7 (46.9) |
|  | Nutrition policy | 2.05 (0.89) | 2.00 (1.15) | 2.84 (1.07) | 58.8 (84.7) | 64.8 (83.5) | 53.3 (89.9) |
| Lunch Box Summary Score | | 1.89 (0.84) | 2.15 (1.07) | 2.31 (1.20) | 22.0 (30.6) | 15.3 (21.9) | 28.1 (36.8) |
|  | Lunch box food/drink provided | 2.36 (0.36) | 2.71 (0.76) | 2.78 (0.60) | 19.8 (27.0) | 10.0 (18.7) | 28.1 (31.7) |
|  | Lunch box education and professional development | 1.68 (1.03) | 2.45 (1.42) | 2.63 (1.46) | 87.3 (124.1) | 74.9 (129.1) | 98.3 (125.3) |
|  | Lunch box policy | 1.50 (0.76) | 1.40 (0.97) | 1.95 (1.35) | 37.7 (96.5) | 18.5 (33.8) | 55.0 (130.1) |
|  | Lunch box feeding environment | 3.62 (0.31) | 3.89 (0.20) | 3.82 (0.19) | 7.1 (13.2) | 0.6 (1.3) | 12.6 (16.4) |
| Physical Activity and Play Summary Score | | 2.71 (0.41) | 3.05 (0.53) | 3.27 (0.52) | 25.3 (31.1) | 16.6 (18.6) | 33.0 (38.6) |
|  | Time provided | 3.25 (0.53) | 3.53 (0.45) | 3.60 (0.39) | 14.2 (25.6) | 18.5 (31.8) | 10.2 (19.4) |
|  | Indoor play environment | 2.98 (0.94) | 3.35 (0.67) | 3.32 (0.84) | 27.0 (65.9) | 3.4 (17.6) | 48.3 (85.6) |
|  | Physical activity staff practices | 3.26 (0.29) | 3.53 (0.32) | 3.61 (0.38) | 11.2 (12.3) | 7.9 (12.7) | 14.1 (11.7) |
|  | Physical activity education and professional development | 2.33 (0.77) | 2.75 (0.92) | 3.16 (1.03) | 59.7 (97.7) | 23.1 (36.3) | 92.6 (124.1) |
|  | Physical activity policy | 1.75 (0.97) | 2.10 (1.29) | 2.68 (1.25) | 97.8 (129.8) | 86.1 (132.9) | 108.3 (133.2) |
| Outdoor Play and Learning Summary Score | | 2.66 (0.48) | 2.98 (0.53) | 3.21 (0.60) | 23.1 (32.8) | 11.3 (30.9) | 33.8 (32.2) |
|  | Outdoor play | 3.48 (0.60) | 3.62 (0.43) | 3.62 (0.42) | 2.6 (14.5) | 1.7 (10.8) | 3.4 (17.8) |
|  | Outdoor physical environment | 3.05 (0.46) | 3.22 (0.45) | 3.20 (0.55) | 5.5 (17.5) | 5.1 (11.3) | 5.8 (22.3) |
|  | Outdoor play education and professional development | 2.27 (0.97) | 2.67 (1.06) | 3.21 (0.77) | 72.8 (92.5) | 25.8 (67.1) | 115.1 (94.5) |
|  | Outdoor play policy | 1.85 (1.09) | 2.40 (1.35) | 2.79 (1.36) | 94.3 (137.2) | 62.0 (137.9) | 123.3 (137.0) |
| Screen Time Summary Score | | 2.21 (0.41) | 2.30 (0.62) | 2.70 (0.80) | 24.9 (34.7) | 22.2 (26.6) | 27.4 (42.0) |
|  | Screen time availability and staff practices | 3.50 (0.49) | 3.55 (0.52) | 3.61 (0.43) | 3.1 (9.4) | -0.3 (8.5) | 6.2 (9.6) |
|  | Screen time education | 1.90 (0.85) | 1.95 (1.17) | 2.55 (1.20) | 48.4 (79.1) | 50.4 (70.0) | 46.7 (90.2) |
|  | Screen time policy | 1.25 (0.55) | 1.40 (0.97) | 1.95 (1.35) | 68.4 (115.7) | 66.7 (111.8) | 70.0 (125.2) |

### Additional file 7: Physical activity and nutrition goal achievement
